# Engineering Versatile Nanomedicines for Ultrasonic Tumor Immunotherapy

**DOI:** 10.1002/advs.202305392

**Published:** 2023-12-02

**Authors:** Jing Liang, Xiaohui Qiao, Luping Qiu, Huning Xu, Huijing Xiang, Hong Ding, Yu Chen

**Affiliations:** ^1^ Department of Ultrasound Huashan Hospital Fudan University Shanghai 200040 China; ^2^ Materdicine Lab School of Life Sciences Shanghai University Shanghai 2000444 China

**Keywords:** immunotherapy, nanomedicine, sonodynamic therapy, tumor therapy, ultrasound technologies

## Abstract

Due to the specific advantages of ultrasound (US) in therapeutic disease treatments, the unique therapeutic US technology has emerged. In addition to featuring a low‐invasive targeted cancer‐cell killing effect, the therapeutic US technology has been demonstrated to modulate the tumor immune landscape, amplify the therapeutic effect of other antitumor therapies, and induce immunosensitization of tumors to immunotherapy, shedding new light on the cancer treatment. Tremendous advances in nanotechnology are also expected to bring unprecedented benefits to enhancing the antitumor efficiency and immunological effects of therapeutic US, as well as therapeutic US‐derived bimodal and multimodal synergistic therapies. This comprehensive review summarizes the immunological effects induced by different therapeutic US technologies, including ultrasound‐mediated micro‐/nanobubble destruction (UTMD/UTND), sonodynamic therapy (SDT), and focused ultrasound (FUS), as well as the main underlying mechanisms involved. It is also discussed that the recent research progress of engineering intelligent nanoplatform in improving the antitumor efficiency of therapeutic US technologies. Finally, focusing on clinical translation, the key issues and challenges currently faced are summarized, and the prospects for promoting the clinical translation of these emerging nanomaterials and ultrasonic immunotherapy in the future are proposed.

## Graphical abstract

This comprehensive review summarizes the immunological effects induced by different therapeutic ultrasound technologies, including ultrasound‐mediated microbubble destruction (UTMD), sonodynamic therapy (SDT), and focused ultrasound (FUS), as well as the main underlying mechanisms involved.

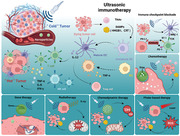

## 1 Introduction

Immune system is extremely complex and involved in the initiation, development, and outcome of various diseases, especially cancer, which is a major cause of morbidity and mortality worldwide.^[^ ^1^, ^2^, ^3^, ^4^, ^5^, ^6^ ^]^ Increased infiltration of suppressive immune cells, including regulatory T cells (Tregs) and myeloid‐derived suppressor cells (MDSCs), polarization of tumor‐associated macrophages (TAMs) from antitumor M1 type to protumor M2 type, incomplete dendritic cell (DC) maturation, tumor‐induced DC dysfunction, and upregulation of programmed cell death protein 1 (PD‐1) upon typical antitumor immune cells, CD8^+^ T lymphocytes co‐producing immunosuppressive tumor microenvironment (TME), limit the antitumor efficiency.^[^ ^5^, ^6^, ^7^, ^8^ ^]^ Therefore, the regulation or reversal of immunosuppressive TME has become a hot spot in tumor therapy.^[^ ^9^, ^10^, ^11^, ^12^ ^]^ In particular, immunotherapy is considered to be a major breakthrough in cancer treatment, such as monoclonal antibodies,^[^ ^13^, ^14^, ^15^ ^]^ cytokine therapy,^[^ ^16^, ^17^, ^18^ ^]^ DC‐based cancer vaccines,^[^ ^19^, ^20^, ^21^ ^]^ immune checkpoint blockade (ICB) therapy against PD‐1, death‐ligand‐1 (PD‐L1), and cytotoxic T lymphocyte antigen‐4 (CTLA‐4),^[^ ^22^, ^23^, ^24^ ^]^ and chimeric antigen receptor (CAR) T‐cell therapy, genetically programming T cells with redirected specificity against malignant cells.^[^ ^24^, ^25^, ^26^ ^]^ In addition, the effects of other antitumor therapies on TME are becoming clear. For example, chemotherapy, as the mainstream of antitumor therapy, can elicit immunogenic cell death (ICD), release damage‐associated molecular patterns (DAMPs), promote DC maturation and the following antitumor responses.^[^ ^27^, ^28^ ^]^ Researches have confirmed that the endoplasmic reticulum (ER) stress and reactive oxygen species (ROS) production are vital constituents in the intracellular mechanisms that control ICD. Simultaneous ER stress and ROS production can increase the diversity of emitted DAMPs, a crucial factor in the immunogenicity of dying cancer cells.^[^ ^29^, ^30^ ^]^ Consequently, the therapeutic efficacy of radiotherapy,^[^ ^31^, ^32^ ^]^ high hydrostatic pressure,^[^ ^33^, ^34^ ^]^ photo‐mediated therapeutic approaches, including photodynamic therapy (PDT)^[^ ^35^, ^36^ ^]^ and photothermal therapy (PTT),^[^ ^37^, ^38^ ^]^ is partially attributed to the induction of ICD.

**Scheme 1:**
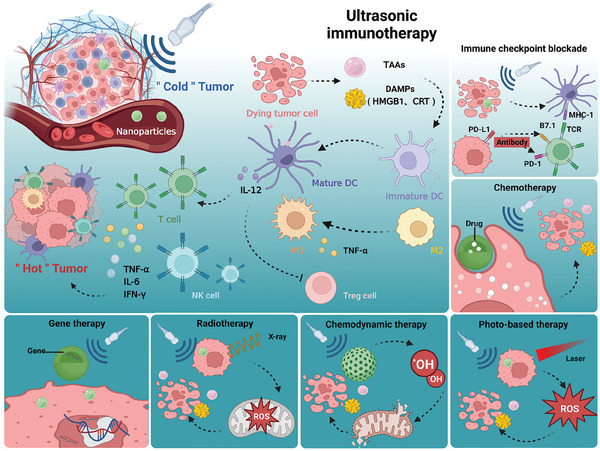
Schematic illustration revealing immunological effects of US, synergistically augmenting the antitumor efficiency with other modalities, including radiotherapy, immunotherapy, chemotherapy, gene therapy, photo‐based therapy, as well as chemodynamic therapy. (Created with bioRender.com)

US is best known for its use in diagnostic imaging. In addition, considering that US is noninvasive, inexpensive, comprehensive diagnosis and treatment, and capable of penetrating deep tissues, and the energy carried by US can affect cell function, it has shown unique advantages in therapeutic applications for tumor treatment. For example, US‐targeted drug/gene delivery technology has become a hotspot of recent research. There is growing evidence that UTMD/UTND can lead to precise release of loaded drug/gene at tumor sites, improve the efficacy of various therapies, and maximize drug‐ or gene‐induced antitumor immune response.^[^ ^39^, ^40^ ^]^ In addition to UTMD/UTND, a number of other therapeutic US techniques have been developed, including SDT and HIFU. Preclinical studies have shown that SDT could utilize sonosensitizers to activate toxic reactive oxygen species (ROS) and elicit cytotoxic events to induce cell/tissue apoptosis and necrosis with minimal invasiveness, which is a promising approach to treat cancer and other diseases.^[^ ^41^, ^42^ ^]^ As a novel type of clinical cancer treatment, HIFU can cause tumor‐cell necrosis and tumor ablation through thermal and mechanical effects. Clinical studies have demonstrated that HIFU has desirable therapeutic efficiency in the treatment of various tumors, including uterine fibroids and pancreas, prostate, breast, liver, kidney, bone and brain tumors.^[^ ^43^, ^44^ ^]^ In addition to the therapeutic evaluation of UTMD/UTND, SDT, and HIFU, their effects on immunity have been studied. The UTMD/UTND‐induced cell lysis, SDT‐induced apoptosis, and HIFU‐induced necrosis can subsequently release tumor‐associated antigens (TAAs) to further activate the antitumor immune response.^[^ ^45^, ^46^, ^47^ ^]^ Based on the rapid development of nanomedicine and nanotechnology, different treatment strategies that combine therapeutic US technologies with other modalities have been proposed,^[^ ^48^, ^49^, ^50^ ^]^ and the immunological effects induced by therapeutic US technologies have the potency to amplify the antitumor immunity of other cancer therapies, transforming “cold” tumors into “hot” ones.^[^ ^49^, ^51^, ^52^ ^]^ Thus, this immunotherapy derived from therapeutic US technologies, that is, ultrasonic immunotherapy, has excellent development prospects (**Scheme** **1**).

## 2 Ultrasound Targeted Micro‐/Nanobubble Destruction

### 2.1 Direct Effect of UTMD/UTND on Immune Response

With the wide application of US and the remarkable development of US contrast agents, the efficacy of UTMD/UTND‐mediated drug/gene delivery has been significantly promoted. Several studies have been performed to shed the light on the underlying mechanism of UTMD/UTND, and it is found that UTMD/UTND mainly depends on cavitation effect, which can induce mechanical force, form the sound holes, and further enhance the permeability of vascular membranes.^[^ ^39^, ^40^ ^]^ In addition, UTMD/UTND has been proven to augment the permeability and retention (EPR) effect, allowing nanomedicine to easily penetrate tumor tissue and remain there for a longer time, thereby improving the efficiency of tumor treatment.^[^ ^40^, ^53^ ^]^

**Figure 1:**
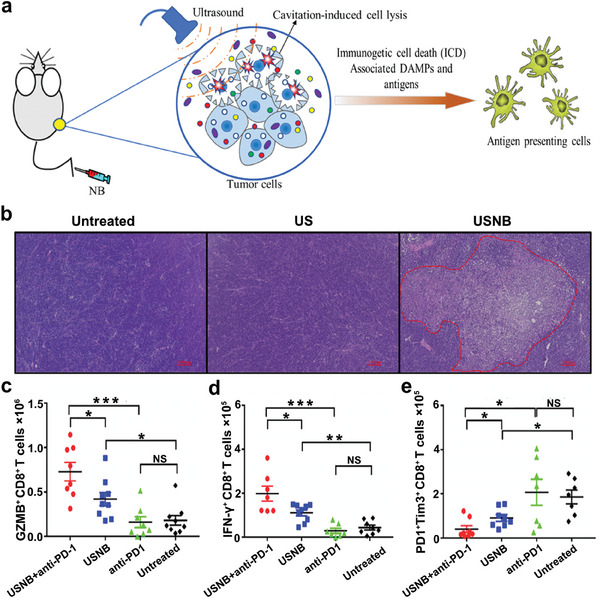
a) Schematic diagram illustrating the impacts of UTNB upon a mouse tumor model. b) H&E staining of tumor tissues after different treatments. c–e) The populations of GZMB^+^CD8^+^ T, IFN‐γ^+^CD8^+^ T, and PD1^+^Tim3^+^CD8^+^ T cells in different groups. Adapted with permission.^[^ ^54^ ^]^ Copyright 2022, BMJ Publishing Group Ltd. * *p*< 0.05, ** *p*< 0.01, *** *p*< 0.001. NS, no significance.

Although delivery mechanism of UTMD/UTND is gradually clarified, the impact of UTMD/UTND on tumor immune microenvironment is still unclear. A study^[^ ^54^ ^]^ found that ultrasound‐stimulated nanobubbles (USNBs) could elicit cavitation effect, and subsequently induce tumor cell lysis with DAMPs releasing. These DAMPs act as “eat me” signals to recruit antigen‐presenting cells (APCs) to present dead cell‐associated antigens to T cells, thereby promote adaptive antitumor immune response, showing great promise in the immunotherapy for cancer treatment (**Figure** **1**a). As shown in Figure 1b, US irradiation (1 MHz, 1 W cm^−2^, 30 s) combined with nanobubbles can directly cause the tumor cell necrosis in vivo, while single US treatment could not. With the activation of APCs, adaptive immune response is triggered, mainly characterized by the upregulation of activated CD8^+^ T cell populations, including granular enzyme B (GZMB) positive CD8^+^ T and interferon‐γ (IFN‐γ) positive CD8^+^ T cells (Figure 1c,d). Furthermore, the expression levels of PD‐1 and T cell immunoglobulin myxin 3 (TIM3), as the markers of T cell exhaustion,^[^ ^7^ ^]^ were analyzed. The results showed that the number of PD1^+^Tim3^+^CD8^+^ T cells decreased after USNB treatment (Figure 1e), implying that USNB could alleviate the exhaustion of CD8^+^ T cells. Since T cell exhaustion is one of the major factors affecting the efficacy of anti‐PD‐1 therapy,^[^ ^55^, ^56^ ^]^ the addition of USNB can further enhance the antitumor immune response induced by anti PD‐1 treatment. This research provides the first empirical evidence that UTMD/UTND can modulate the tumor microenvironment without the assistance of drug delivery, thereby unveiling the therapeutic immunomodulatory potential of US. This holds substantial implications for guiding subsequent related studies.

### 2.2 Indirect Synergistic Effect of UTMD/UTND on Immunotherapy

#### 2.2.1 Promoting Chemotherapy‐Induced Antitumor Immune Response

**Figure 2:**
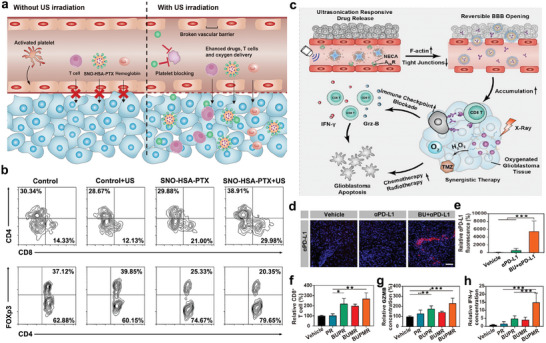
a) Schematic illustration revealing the effects of SNO‐HSA‐PTX upon tumor vascular barrier. b) Flow cytometry analysis of intra‐tumoral CD4^+^ T, CD8^+^ T, and Treg (Foxp3^+^CD4^+^) cell infiltration. Reproduced with permission.^[^ ^60^ ^]^ Copyright 2021, Dove Medical Press. c) Schematic diagram of targeted regulation of the blood‐brain barrier to improve the efficiency of chemoradiotherapy and immune checkpoint blocking in the treatment of glioblastoma. d,e) The accumulation of PD‐L1 in glioblastoma tissues was analyzed by immunofluorescence. f,g) The infiltration of CD8^+^ T cells in glioblastoma tissues after various treatments. g,h) The levels of GZMB and IFN‐γ analyzed by ELISA in different groups. Reproduced with permission.^[^ ^76^ ^]^ Copyright 2021, American Chemical Society. * *p*< 0.05, ** *p*< 0.01, *** *p*< 0.001.

Since UTMD/UTND has been widely used in targeted drug/gene delivery therapy, UTMD/UTND has an indirect synergistic effect on antitumor immune response. First, given the resistance and systematic toxicity of chemotherapy, great efforts have been devoted to investigating UTMD/UTND‐mediated targeted chemotherapy to minimize side effects and augment therapeutic efficacy.^[^ ^57^, ^58^, ^59^, ^60^ ^]^ Study has revealed that UTND‐mediated inertial cavitation activity (1.1 MHz) could promote synergy with chemotherapy via causing tumor necrosis in a 3D model of pancreatic ductal adenocarcinoma. Part of the reason for tumor suppression may be attributed to the fact that tumor necrosis could activate ICD‐induced antitumor immune response.^[^ ^59^ ^]^ In addition, as shown in **Figure** **2**a, nitric oxide (NO) release nanoparticles, SNO‐HSA‐PTX, loading chemotherapeutic drug, paclitaxel (PTX), could release NO under US irradiation, inducing the blockade of platelets in tumor‐bearing mice. This caused the damage of vascular barrier, thereby enhancing the delivery of drugs, T cells, as well as oxygen. The combination of SNO‐HSA‐PTX and US treatment (1 MHz, 2 W cm^−2^, 5 min) could significantly suppress the tumor growth of breast cancer mice by promoting the infiltration of intra‐tumoral CD4^+^/CD8^+^ T cells, and downregulating the population of Treg cells (Figure 2b). Given the inhibitory effect of US‐triggered NO‐releasing nanoparticles on platelets at tumor sites, this study also assessed the impact of SNO‐HSA‐PTX + US on the normal hemostatic function of platelets, and the results reveled that SNO‐HSA‐PTX+US treatment did not significantly influence the bleeding time in mice, underscoring the safety of SNO‐HSA‐PTX for in vivo cancer treatment.^[^ ^60^ ^]^

Furthermore, alleviating tumor hypoxia may be another reason for the excellent antitumor effect, as hypoxic tumor environment may limit the activity of immune cells, promote malignant transformation, migration, and poor tumor prognosis.^[^ ^61^, ^62^, ^63^, ^64^ ^]^ Studies have demonstrated that that US‐targeted microbubble cavitation could effectively increase tumor hemoperfusion, alleviate tumor hypoxia, and enhance effectiveness of radiotherapy.^[^ ^65^ ^]^ This enhancement is facilitated by purinergic signaling and the discharge of NO through the activation of endothelial nitric oxide synthase.^[^ ^66^ ^]^ Given the widespread use of contrast US agents in clinic, this technology is expected to improve the effectiveness of other treatment modalities, such as chemotherapy and immunotherapy. This suggests that US‐targeted microvesicle cavitation exhibits potentially transformative effects in future cancer treatment. Additionally, UTMD/UTND‐mediated oxygen delivery plays a crucial role in tumor therapy. For instance, an oxygen‐carrying RBC‐PFC nanoemulsion, combined with AuNW nanomotor, was built to penetrate the cell membrane and conduct intracellular oxygen transfer in macrophages under US irradiation (6 V, 2.66 MHz, 5 min), which successfully improved the survival rates of macrophages.^[^ ^67^ ^]^ As macrophages are a critical APC, they present TAAs to effector cells, which might further enhance the following antitumor immune response. Also, this oxygen‐delivery platform can be expanded to other immune cells to boost their activity.

#### 2.2.2 Promoting ICB‐Induced Immunotherapy

Although ICB‐induced immunotherapy has great benefits for cancer patients, there are increasing reports of immune‐related adverse effects and allergic reactions.^[^ ^68^, ^69^, ^70^, ^71^ ^]^ Hence, UTMD/UTND‐mediated targeted transport of antibodies may alleviate the xenotoxicity and maximize the therapeutic efficacy. According to this principle, a new microbubble labeled with PD‐1 antibody was developed for the treatment of the mice bearing CT26 colon carcinoma. The results showed that such immune‐microbubble complex could enhance the localization of PD‐1 antibody in tumor tissues and significantly augment the suppression of tumor growth.^[^ ^72^ ^]^ Additionally, there is evidence that UTMD/UTND facilitates the temporary increase of blood‐brain barrier (BBB) permeability to enhance drug delivery to the brain parenchyma, so that it is possible to perform immunotherapy for cerebral diseases, particularly glioblastoma, as the most fatal brain cancer.^[^ ^73^, ^74^, ^75^, ^76^ ^]^ BBB‐regulating nanovesicles (BRN) loaded with adenosine 2A receptor agonists were developed to achieve targeted BBB regulation by releasing 2A receptor agonists in the presence of ultrasonication (0.5 W cm^−2^, 3 min), further inducing the tight connection between F‐actin and endothelial cells (Figure 2c). Figure 2d,e shown that PD‐L1 antibody alone could not efficiently enter the orthotopic glioblastoma, thereby leading to the clinical failure of anti‐PD‐L1 therapy for glioblastoma. Furthermore, compared to other treatment groups, BRN could assist anti‐PD‐1 therapy and radiotherapy to stimulate stronger antitumor immunity, upregulate the infiltration of CD8^+^ T cells in tumors (Figure 2f), and promote the activation of CD8^+^ T cells with increased GZMB and IFN‐γ secretion (Figure 2g,h).^[^ ^76^ ^]^

With the deepening of the understanding of tumor immunity, the researchers found that in addition to established targets such as PD‐1, PD‐L1, CTLA‐4, new targets such as CD300ld,^[^ ^77^ ^]^ discoid domain receptor tyrosine kinase 1 (DDR1),^[^ ^78^ ^]^ and V‐domain immunoglobulin suppressor of T cell activation (VISTA)^[^ ^79^ ^]^ also play an important role in tumor immunosuppression. Developing antibodies against these new markers could further revolutionize tumor immunotherapy. While the use of nanomaterials in this context is still in its infancy, there is a growing belief that US‐mediated drug delivery, similar to BRN nanoparticles that improve anti‐PD‐L1 therapy, can improve the efficacy of these targeted antibodies in treating cancer.

#### 2.2.3 Promoting Tumor Vaccine‐Induced Antitumor Immunity

Tumor vaccine is a promising therapeutic strategy, which can trigger the patients' autoimmune system to target and eliminate tumor tissues. In particular, DC vaccine has been proved to have made great progress in antitumor immunotherapy^[^ ^19^, ^20^, ^21^ ^]^ and is considered as an effective therapeutic strategy for tumor metastasis and recurrence.^[^ ^80^ ^]^ In this treatment strategy, it is important for DC to present abundant epitopes from TAAs via major histocompatibility complex (MHC) class I and MHC class II molecules.^[^ ^81^ ^]^ Thus, the combination of bubble liposomes and US treatment (2 MHz, 2.0 W cm^−2^, 3 × 10 s) was proposed to deliver melanoma‐derived antigen into DCs, inducing antigen‐specific cytotoxic T lymphocytes (CTLs), and found that this treatment strategy can significantly prevent lung metastasis of melanoma.^[^ ^82^ ^]^

**Figure 3:**
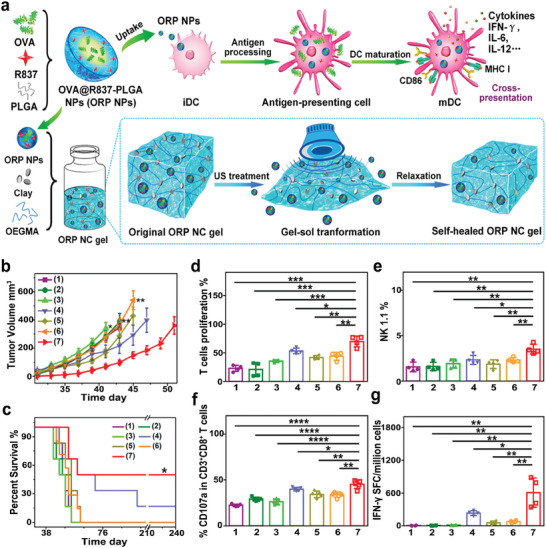
a) The diagram depicting the composition of nanovaccine ORP NPs and US‐responsive release of OPR NPs from the self‐healing gel system. b) The curves of tumor growth in different groups. c) Survival analyses of mice after various treatments. d–g) Analyses of splenic T cell proliferation, NK cells percentage, CD107a‐postive CD3^+^CD8^+^ T cells percentages and the number of IFN‐γ spot‐forming cells in splenocytes. Groups are allocated as follow: 1) Control group, 2) Blank NC group, 3) Free ORP (single injection) group, 4) Free ORP (multiple injection) group, 5) ORP NC gel group, 6) ORP NC gel + single US treatment group, and 7) ORP NC gel + multiple US treatments group. Reproduced with permission.^[^ ^85^ ^]^ Copyright 2021, American Chemical Society. * *p*< 0.05, ** *p*< 0.01, *** *p*< 0.001.

In addition, the presence of immunogenic adjuvants can potentiate the efficacy of immunotherapy, including tumor vaccines.^[^ ^83^ ^]^ Thus, ORP NPs was designed, which consisted of tumor antigen ovalbumin (OVA) and immune adjuvant, imiquimod (R837). As multiple stimulations by repeated administrations of nanovaccines are often required for optimal therapeutic efficacy, this may reduce patient compliance and increase the risk of side effects.^[^ ^84^ ^]^ To address this issue, this study innovatively loaded OPR NPs in hydrogel system to construct an US‐responsive nanovaccine. This nanovaccine system could be transformed into a sol state under ultrasonic irradiation, allowing for the release of the nanovaccines, and then recover to a gel state after getting rid of ultrasonic stimulation (**Figure** **3**a). Based on this system, a single subcutaneous injection of nanovaccine‐loaded gel and multiple US treatments (40 kHz, 6 W cm^−2^, 50% duty cycle, 5 min) could effectively inhibit tumor growth and significantly improve the survival rates of mice (Figure 3b,c). Meanwhile, the treatment promoted the proliferation of T cells, facilitated the activation of NK cells and CD8^+^ CLTs (CD3^+^CD8^+^CD107a), as well as upregulated the number of IFN‐γ spot‐forming cells in the spleen (Figure 3d–g).^[^ ^85^ ^]^ As vaccines are one of utmost important weapons for modern medicine to fight against various diseases, this unique nanovaccine‐loaded gel would change the current situation of repeated vaccine injections and provide new strategies for cancer treatment.

#### 2.2.4 Promoting Antitumor Immunity Induced by Gene Therapy

**Figure 4:**
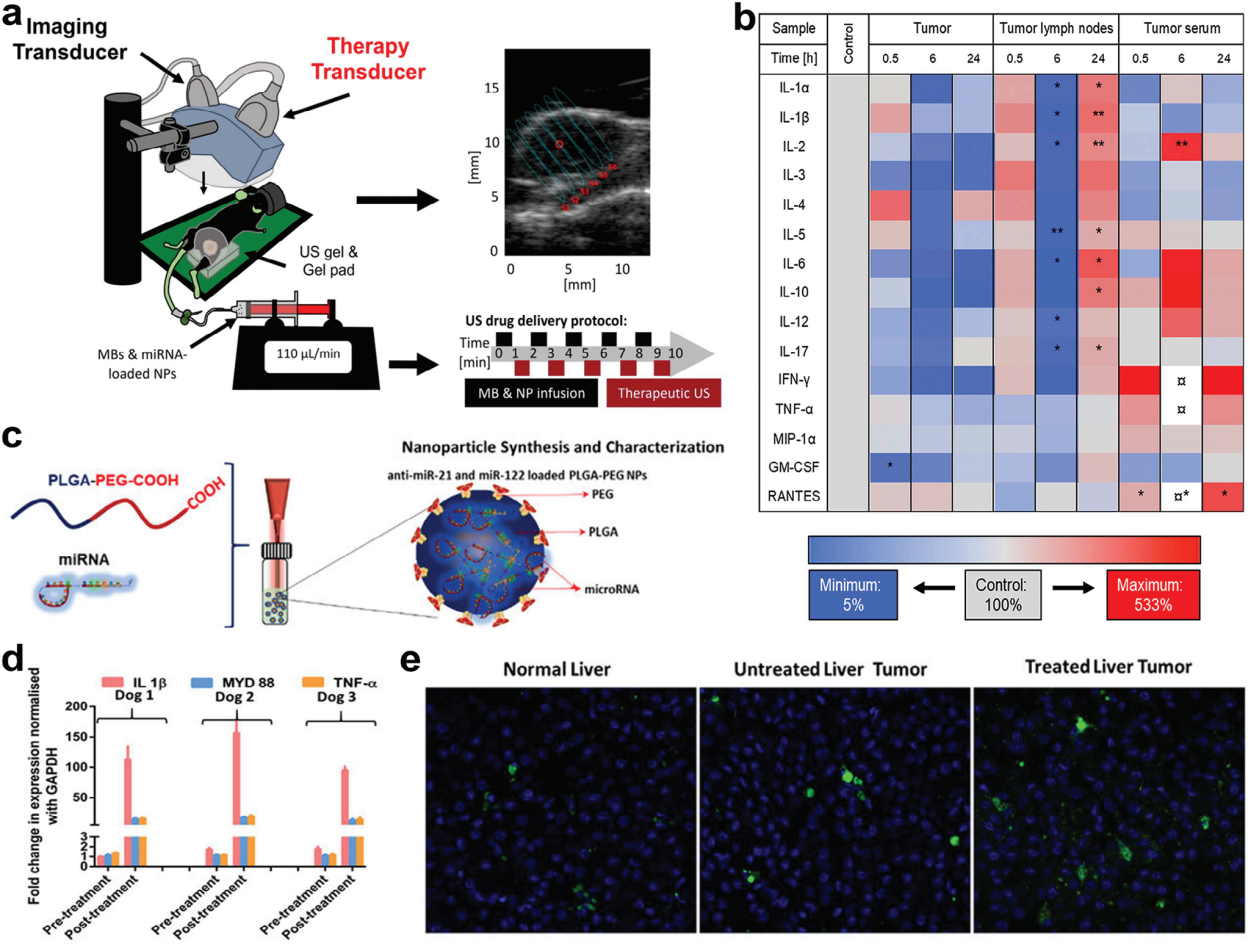
a) Schematic of experimental procedure. b) Heatmap displaying the expression changes of cytokines in tumors, tumor lymph nodes and serum after the therapy. The sign (¤) indicates that the expression level was higher than the maximum. Reproduced with permission.^[^ ^92^ ^]^ Copyright 2020, Elsevier. c) Schematic illustration of microRNA‐loaded PLGA‐b‐PEG NPs synthesis. d) Analysis of inflammatory cytokine markers in canine blood before and after US‐guided microRNA therapy. e) Immunofluorescence analysis of CD8^+^ T cell infiltration in normal liver, untreated liver tumor and treated liver tumor. Reproduced with permission.^[^ ^94^ ^]^ Copyright 2020, John Wiley and Sons.

With the development of genetic engineering and the gradual elucidation of tumor pathogenesis, gene therapy has shown great prospects in the effective treatment of tumors.^[^ ^86^, ^87^ ^]^ Recently, enormous efforts have been made to develop gene delivery nanomaterials. Because microRNA‐122 has been shown to inhibit tumor progression,^[^ ^88^, ^89^ ^]^ and microRNA‐21, which is highly expressed in hepatocellular carcinoma (HCC), could promote tumor cell proliferation and migration,^[^ ^90^, ^91^ ^]^ a study delivered microRNA‐122 and anti‐microRNA‐21 to mouse HCC model via UTMD (1.8 MHz, 10 min) to investigate the changes in the immune microenvironment (**Figure** **4**a). The results revealed that the combination of microRNA and UTMD regulated the overall positive treatment response through the downregulation of pro‐tumor cytokines (Figure 4b).^[^ ^92^ ^]^

However, this study only analyzed the cytokine levels and did not confirm the effect of this combination therapy on immune cell populations. Furthermore, PLGA‐b‐PEG NPs, loaded with microRNA‐122 and anti‐microRNA‐21 (Figure 4c), were proved to not only upregulate the antitumor cytokines, including interleukin‐1β (IL‐1β), MYD 88 (an adaptor molecule in the Toll‐like receptor signaling pathway required for activating CD8^+^ T cells),^[^ ^93^ ^]^ and tumor necrosis factor‐α (TNF‐α; Figure 4d), but also induce the infiltration of CD8^+^ T cells in tumor region of the canine HCC model to reverse the immunosuppressive TME under US irradiation (2 MHz, 2 min, Figure 4e).^[^ ^94^ ^]^ Since the occurrence, pathology and genetics of human HCC are similar to those in dogs, and mature microRNA are human replicas, the results obtained in this study may be translated for HCC patients. For gene therapy, nanoparticles have received significant interest due to their unique size‐dependence, accessible alteration methods, and capacity to efficiently guide targeted gene delivery. Advances in US‐mediated gene delivery are anticipated to offer substantial promise for the clinical translation of gene therapy.

## 3 Sonodynamic Therapy

### 3.1 SDT‐Guided Monotherapy

#### 3.1.1 Inducing ICD‐Based Antitumor Immune Response

**Figure 5:**
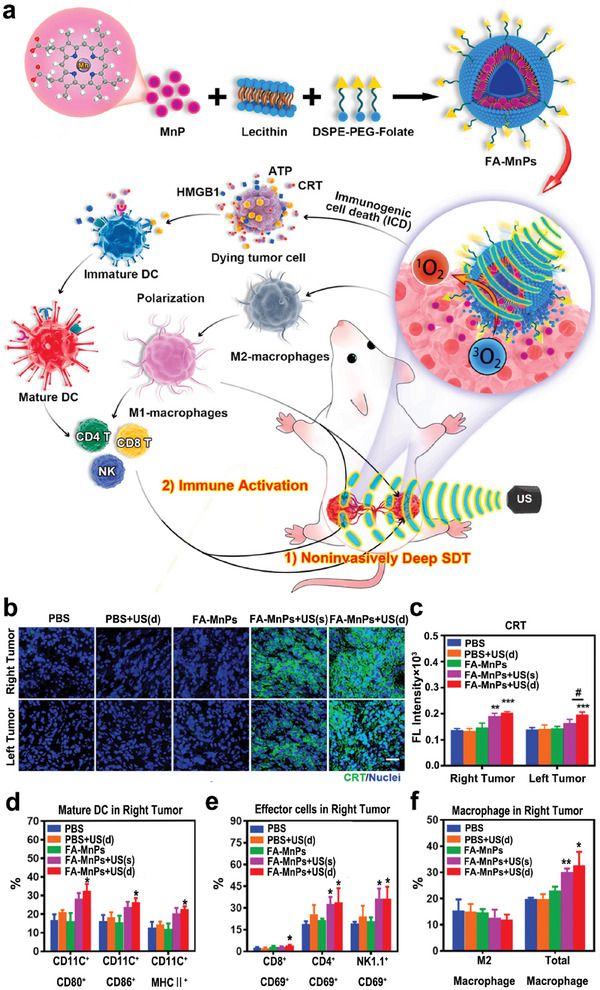
a) Diagram depicting SDT treatment and immune activation mediated by FA‐MnPs. b,c) Immunofluorescence analysis of CRT expression in 4T1 tumors post different treatments, including PBS group, PBS + US group with depth‐penetrating US treatment from right to left, FA‐MnPs group without US, FA‐MnPs + US(s) group with only right tumor treated by US, and FA‐MnPs + US(d) group with US penetration treatment from the right tumor to the left. d–f) The analyses of mature DCs, effector cells (activated CD8^+^ T, CD4^+^ T, and NK cells) and macrophages infiltration in right tumors. Reproduced with permission.^[^ ^45^ ^]^ Copyright 2021, Elsevier. * *p*< 0.05, ** *p*< 0.01, *** *p*< 0.001 versus PBS group; # *p*< 0.05 for FA‐MnPs(d) group versus FA‐MnPs(s) group.

Specifically, SDT refers to sonochemical events that depend on the sonosensitizers in the sound field. In the presence of molecular oxygen, the sonosensitizers produce singlet oxygen (^1^O_2_) or hydroxyl radical (·OH) under US irradiation, resulting in strong intracellular cytotoxicity. The mechanism of ROS generation during SDT remains elusive. Some plausible explanations are based on cavitation effect, including sonoluminescence and pyrolysis.^[^ ^48^, ^95^ ^]^ The interplay between ER stress and ROS production forms the basis of the SDT mechanism. This therapy harnesses the cytotoxicity of ROS and the cellular response to ER stress to induce ICD, leading to the surface exposure of calreticulin (CRT), a protein in the endoplasmic reticulum, which maintains Ca^2+^ homeostasis.^[^ ^96^ ^]^ For instance, a multifunctional nanosonosensitizer (FA‐MnP) was designed by encapsulating an organic sonosensitizer, manganese‐protoporphyrin (MnP), in folate‐liposomes to implement SDT. A bilateral tumor‐bearing mouse model was built, and the right tumors, as the superficial tumors, were treated with US irradiation (1.0 MHz, 2.0 W cm^−2^, 5 min, 50% duty cycle), while the left ones were considered as the deep‐seated tumors (**Figure** **5**a). Multifunctional FA‐MnPs could suppress the growth of superficial and deep‐seated tumors simultaneously, indicating that FA‐MnPs‐mediated SDT has good tissue penetration. In addition, FA‐MnPs‐mediated SDT induced ICD in bilateral tumors with the increase of CRT (Figure 5b,c). Flow cytometry analysis revealed that FA‐MnPs‐mediated SDT could reverse the immunosuppressive TME in the right tumors, promote the maturation of DCs, polarize immunosuppressive M2 into antitumor M1 macrophages, and activate T cells and NK cells (Figure 5d–f). Similar results were observed in the draining lymph nodes and untreated left tumors. After treatment with FA‐MnPs and subsequent US actuation, the percentage of Treg cells in draining lymph nodes was downregulated. These results implied that FA‐MnPs‐mediated SDT might elicit a systemic antitumor immunity.^[^ ^45^ ^]^

Based on the tumor hypoxia relief and high oxygen loading capacities of perfluorocarbons (PFC) based oxygen nanoparticles, fluorine‐containing covalently conjugated polymers (COPs), denoted as PFCE@THPP*_pf_*‐COPs, were fabricated by conjugating organic sonosensitizer, meso‐5, 10, 15, 20 tetra‐(4‐hydroxyphenyl) porphyrin (THPP), perfluoroacetic acid (PFSEA), and mPEG5k‐COOH through the esterification reaction. After intratumoral injection, PFCE@THPP*_pf_*‐COPs relieved tumor hypoxia significantly, and thus efficiently inhibited tumor growth under US actuation (40 kHz, 2 W, 30 min) by inducing ICD of cancer cells. Furthermore, the combination of SDT and anti‐CD47 immunotherapy could synergistically suppress tumor proliferation by increasing intratumoral infiltration of CD3^+^CD8^+^ T cells and reducing the frequency of immunosuppressive Tregs.^[^ ^97^ ^]^ These might all be attributed to ICD‐induced antitumor responses.

#### 3.1.2 Potentiating ICD‐Based Antitumor Immune Response

**Figure 6:**
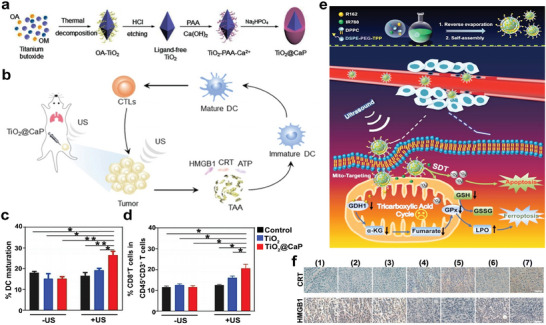
a) Schematic depiction of the composition of TiO_2_@CaP nanoparticles. b) Diagram depicting the mechanism of TiO_2_@CaP‐mediated immunotherapy. c,d) Quantification of DC maturation in lymph nodes and CD3^+^CD8^+^ T cells in 4T1 tumors after different treatments. Reproduced with permission.^[^ ^98^ ^]^ Copyright 2021, John Wiley and Sons. e) Diagram of synthetic MLipRIR NPs and the effect of MLipRIR NPs on redox homeostasis, under the irradiation of US. f) Immunohistochemical analysis of CRT and HMGB1 expressions in tumor harvested on day 14. Groups are allocated as follow: (1) Control group, (2) US group, (3) MLipR + US group, (4) MLipIR + US group, (5) LipRIR + US group, (6) MLipRIR group, and (7) MLipRIR + US group. Adapted with permission.^[^ ^100^ ^]^ Copyright 2021, IVYSPRING. * *p*< 0.05, ** *p*< 0.01.

Although SDT has played a crucial role in suppressing tumor growth and promoting antitumor immune response, this immune response induced by SDT may not be sufficient to translate into a long‐term antitumor immune environment to completely inhibit tumor growth.^[^ ^97^, ^98^ ^]^ To enhance ICD and ICD‐induced antitumor immunity, a transformable nanosonosensitizer (TiO_2_@CaP) was designed, which simultaneously reinvigorated ROS generation and induced Ca^2+^ release in an acidic TME (**Figure** **6**a). Since Ca^2+^ overload could trigger mitochondrial dysfunction,^[^ ^99^ ^]^ the acid‐activated Ca^2+^ ion release of TiO_2_@CaP led to a substantial accumulation of Ca^2+^ ions, which thus accelerated the production of ROS. To synergize with SDT (US parameters: 3 MHz, 2.1 W, 20 min) in the TME, TiO_2_@CaP could significantly promote tumor‐cell apoptosis and further amplify ICD‐induced antitumor immune response (Figure 6b). Compared with TiO_2_ alone, TiO_2_@CaP‐mediated SDT had a higher percentage of DC maturation and more infiltration of CD3^+^CD8^+^ T cells in 4T1 tumors, resulting in better antitumor efficiency (Figure 6c,d).^[^ ^98^ ^]^

In addition, cancer cells often exhibit special defects in performing cell death due to the resistance mechanisms of innate or evolutionary apoptosis, which may inhibit SDT‐mediated apoptosis of tumor cells. A growing number of studies are focused on targeting non‐apoptotic pathways that regulate cell death to improve the efficiency of cancer treatment.^[^ ^100^, ^101^, ^102^, ^103^ ^]^ Hence, mitochondrial‐targeting liposomal nanoparticles (termed as MLipRIR NPs) were synthesized via encapsulating the inhibitor of glutamate dehydrogenase 1 (GDH1), R162, and sonosensitizer, IR780, into a lipid bilayer (Figure 6e). Under US irradiation (1 MHz, 1.5 W cm^−2^, 50% duty cycle), MLipRIR NPs not only released R162 to cause imbalance of redox homeostasis, but also generated ROS, which simultaneously induced severe ferroptosis, a novel type of cell death different from other apoptotic and non‐apoptotic death forms,^[^ ^103^ ^]^ and apoptosis of tumor cells. This synergistic ferroptosis and apoptosis effectively augmented ICD. The increased CRT increased after treatment with MLipRIR and US irradiation. Besides, the release of high mobility group box 1 (HMGB1), a nuclear protein responsible for DNA organization and transcriptional regulation, from the nuclear region to the extracellular space is considered as the late event of ICD.^[^ ^24^ ^]^ Immunohistochemical staining showed significant HMGB1 release after treatment with MLipRIR and US actuation (Figure 6f). Following ICD, the DAMPs release promoted DC maturation and CTL activation, and triggered rapid inflammatory activation with the upregulation of TNF‐α, IL‐6 and IFN‐γ levels in serum, subsequently leading to immune‐mediated tumor suppression.^[^ ^100^ ^]^ Furthermore, a nanoplatform (RSL_3_@O_2_‐ICG NB) loaded with RAS‐selective lethality (RSL3, ferroptosis promoter), was constructed to enhance SDT and promote ferroptosis. RNA‐Seq data exhibited that after the treatment of RSL_3_@O_2_‐ICG NB and US irradiation (1.0 W cm^−2^, 30 s), the differentially expressed genes significantly enriched in ferroptosis‐related pathway. It has been demonstrated that this two‐in‐one nanoplatform could regulate the proportion of immune cells in the TME and further improve the sensitivity of ferroptosis.^[^ ^104^ ^]^ Pyroptosis is a newly identified form of programmed cell death that shows promise in tumor immunotherapy due to its ability to activate innate immune response.^[^ ^105^ ^]^ One study reported an effective strategy based on a sonosensitizer that involved loading LY364947 (a selective inhibitor of transforming growth factor‐β type I receptors chosen for extracellular matrix normalization) into a porous coordination network (PCN‐224) camouflaged with a red blood cell membrane. The efficient SDT (US parameters: 1.0 MHz, 0.5 W cm^−2^, 50% duty cycle, 10 min)‐induced pyroptosis led to upregulation of caspase‐3‐mediated gasdermin E expression at N‐terminal fragments. This stimulated DC maturation and intratumoral T‐cell infiltration, thereby inhibiting tumor growth, facilitating the formation of immunological memory, and prolonging survival.^[^ ^106^ ^]^ Thus, inducing ferroptosis or pyroptosis shows great attraction in inhibiting tumor growth and may provide a therapeutic strategy for the application of other non‐apoptotic pathways in tumor treatment. Although these non‐apoptotic pathways are currently limited to basic research, they have clinical translational potential to innovate the treatment of cancer patients.

Additionally, there is evidence that autophagy, primarily as a tumor initiator, may demonstrate resistance to tumor‐cell apoptosis by providing nutrients for energy supply in advanced cancer.^[^ ^107^, ^108^ ^]^ Thus, nanoplatforms with autophagy inhibitors can optimize the therapeutic effect of SDT. Such as CCM‐HMTNPs/HCQ, a nanoplatform based on hollow mesoporous titanium dioxide nanoparticles, loaded with the autophagy inhibitor, hydroxychloroquine (HCQ) sulfate, and coated with cancer cell membranes, could make breast cancer cells more sensitive to SDT, leading to a better therapeutic effect of SDT (US parameters: 1 W cm^−2^, 30 s).^[^ ^109^ ^]^ Then, ICD and ICD‐based antitumor immune response may also be enhanced by introduction of the autophagy inhibitors. Based on the aforementioned studies, the strategic creation of innovative sonosensitizers with efficient sonosensitization and TME modulation capabilities marks a promising research trajectory for achieving highly effective tumor suppression. This is due to their pronounced ability to initiate the host's antitumor immunity. Furthermore, given their synergistic potential in enhancing the host's antitumor immune response, in‐depth exploration of combination therapies involving immunogenic SDT and various forms of immunotherapy is essential.

### 3.2 SDT‐Derived Bimodal Synergistic Therapy

#### 3.2.1 Dual‐Modality Synergistic Therapy to Enhance ICD‐Induced Antitumor Immune Response

**Figure 7:**
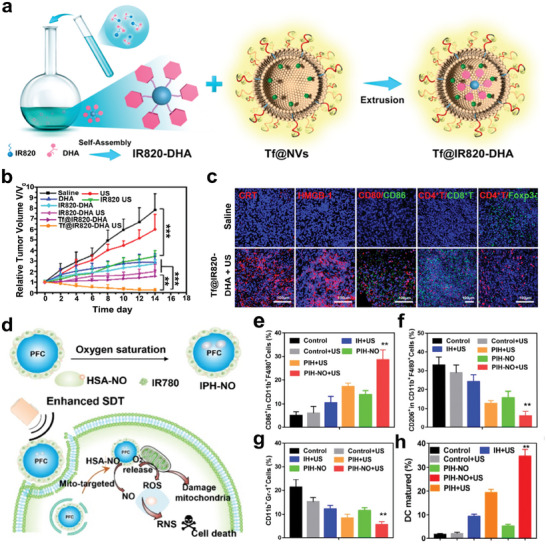
a) Schematic illustration of the composition of Tf@IR820‐DHA. b) The changes of tumor volume in mice with different treatments. c) Immunofluorescence staining of DAMPs (CRT and HMGB1), immune infiltration (CD80/CD86, CD4^+^ T/CD8^+^ T, and CD4^+^ T/Foxp3^+^) in tumor tissues. Reproduced with permission.^[^ ^111^ ^]^ Copyright 2022, American Chemical Society. d) A scheme illustrating the combination therapy of SDT and gas therapy using PIH‐NO nanosystem. e–h) The proportion of M1 (CD11c^+^F4/80^+^CD86^+^), M2 (CD11c^+^F4/80^+^CD206^+^), MDSCs (CD11b^+^Gr‐1^+^) and mature DCs (CD11b^+^CD86^+^CD80^+^) according to flow cytometry analysis. Adapted with permission.^[^ ^114^ ^]^ Copyright 2021, IVYSPRING. ** *p*< 0.01, *** *p*< 0.001.

Given the limited half‐life and diffuse range of ROS, SDT may not be sufficient to effectively and completely suppress tumor growth and metastasis.^[^ ^110^ ^]^ There is evidence that in addition to SDT, immunogenic chemotherapy, radiotherapy, chemodynamic therapy (CDT), PDT, and PTT could induce intracytoplasmic reticulum stress and trigger ICD‐induced antitumor immune response. Therefore, numerous efforts have been devoted to developing nanoplatforms that combine SDT with other modalities to amplify ICD‐mediated antitumor immune response and synergistically treat cancer. Based on this, Tf@IR820‐DHA nanoparticles were fabricated by encapsulating the sonosensitizer (IR820) and dihydroartemisinin (DHA) into transferrin‐expressing cell membrane nanovesicles. The designed Tf@IR820‐DHA nanoparticles could not only specifically target tumor tissues with high expression level of the transferrin receptor (TfR1), but also combine CDT with SDT to promote the generation of ROS and cause a high level of targeted ICDs (**Figure** **7**a).^[^ ^111^ ^]^ Studies have demonstrated that DHA is not only an anticancer drug that produces ROS, but also an efficient ICD inducer.^[^ ^112^, ^113^ ^]^ The results showed that the combination of IR820‐mediated SDT and DHA‐mediated CDT had the best therapeutic efficiency compared to other treatment groups (Figure 7b). After treated with Tf@IR820‐DHA and US actuation (0.4 W cm^−2^, 5 min), the levels of ICD indicators increased, including CRT and HMGB1, followed by a significant upregulation of CD4^+^ T and CD8^+^ T cell infiltration in TME, as well as a sharp decrease in the proportion of Treg cells (Figure 7c). Additionally, a PIH‐NO nanosystem consisted of a human serum albumin‐based NO donor (HSA‐NO) encapsulating perfluorodecalin (FDC) and a sonosensitizer (IR780), could achieve the synergistic treatment of NO‐mediated gas therapy and SDT (Figure 7d). Under US irradiation (1.0 MHz, 1 W cm^−2^, 5 min), PIH‐NO relieved hypoxia and amplified ICD‐induced immune response, and the proportion of M1 and mature DC in tumor tissues was upregulated. Furthermore, the percentages of critical drivers of immunosuppressive TME, including M2 and MDSCs, were also significantly decreased, ultimately reversing immunosuppressive TME and enhancing antitumor efficacy (Figure 7e–h).^[^ ^114^ ^]^

Inspired by photo‐mediated therapeutic approaches, a difunctional sono‐/photo‐sensitizer, amphiphilic rose bengal (RB) nanocapsules, was used in the combination therapy of SDT (US parameters: 1.0 MHz, 1.0 W cm^−2^, 50% duty cycle, 3 min) and PDT (laser parameters: 808 nm, 1.5 W cm^−2^, 1.5 cm, 3 min). The synergistic treatment promoted ROS production, leading to better efficacy in suppressing tumor growth and significantly increased levels of TNF‐α and IL‐6, which indicated cancer‐cell necrosis and a positive prognosis.^[^ ^115^ ^]^ In addition, a novel Pt‐CuS Janus composed of hollow semiconductor CuS and noble metallic Pt, was successfully fabricated. The hollow structure of CuS with a large inner cavity was loaded with sonosensitizer molecules, tetra‐(4‐aminophenyl) porphyrin (TAPP), for implementing SDT (US parameters: 1.0 MHz, 1.0 W cm^−2^, 60% duty cycle, 5 min) and PTT (808 nm, 1.0 W cm^−2^, 7 min). This treatment strategy achieved almost complete tumor suppression under the guidance of photoacoustic and near‐infrared thermal imaging, which provides a new paradigm for the design of multifunctional nanomaterials with integrated diagnosis and treatment functions. Throughout the treatment, parameters including the body weights of the mice, blood indices (such as alanine aminotransferase, alkaline phosphatase, aspartate aminotransferase, blood urea nitrogen and creatine) as well as H&E staining of the main organs collectively suggested that no significant damage occurred.^[^ ^116^ ^]^ Since both PDT and PTT can elicit ICD, the excellent therapeutic effect of combination of PDT/PTT and SDT may be partly attributed to the augmented ICD‐induced antitumor immune response.

**Figure 8:**
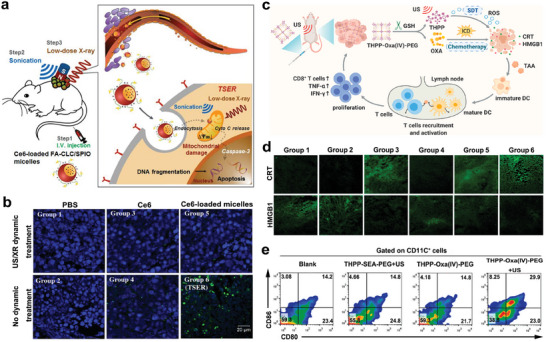
a) Schematic illustration of tumor cell targeted radiotherapy using CE6‐loaded FA‐CLC/SPIO micelles to promote tumor cell apoptosis. b) DNA fragmentation of tumor sections in mice with various treatments. Green fluorescence is an indicator of apoptotic cells. Groups are allocated as follow: (1) PBS group, (2) PBS + US/XR group, (3) Ce6 group, (4) Ce6 + US/XR group, (5) Ce6‐loaded micelles group and (6) Ce6‐loaded micelles + US/XR (TSER) group. Reproduced with permission.^[^ ^118^ ^]^ Copyright 2016, Elsevier. c) Diagram illustrating the mechanism of THPP‐Oxa (IV)‐PEG‐mediated immunotherapy. d) Confocal fluorescence images of CRT and HMGB1 in tumor sections of CT26 tumor bearing mice after different treatments. Groups are allocated as follow: (1) Blank group, (2) US group, (3) Oxaliplatin group, (4) THPP‐SEA‐PEG + US group, (5) THPP‐Oxa (IV)‐PEG group and (6) THPP‐Oxa (IV)‐PEG + US group. e) The analysis of DC maturation in the lymph nodes adjacent to the tumors in different groups. Reproduced with permission.^[^ ^119^ ^]^ Copyright 2022, Elsevier.

Radiotherapy is one of the well‐known cancer treatment strategies, however, the tolerance of normal tissues towards radiotherapy may restrain its therapeutic effectiveness.^[^ ^117^ ^]^ Therefore, the combination of SDT and radiotherapy has been proposed to improve the therapeutic efficacy. As shown in **Figure** **8**a, folic acid‐conjugated carboxymethyl lauryl chitosan/superparamagnetic iron oxide (FA‐CLC/SPIO) micelles were synthesized to efficiently deliver the sonosensitizer, chlorin e6 (Ce6), for targeted sensitization enhanced radiotherapy (TSER). In vivo therapeutic evaluation showed that TSER treatment (US parameters: 1 MHz, 1 W cm^−2^, 20% duty cycle, 10 min; X‐ray parameters: 2 Gy, 6 MV, 30 min) had the best tumor inhibition efficacy among all the treatment groups by promoting tumor cell apoptosis (Figure 8b). Since ICDs can be triggered by radiotherapy, this TSER strategy may amplify the ICD‐induced antitumor immune response.^[^ ^118^ ^]^

Additionally, tremendous endeavors have been made to combine chemotherapy with SDT. To realize more efficient SDT, organic sonosensitizer, THPP, was esterified with carboxyl group terminated PEG and succinic acid conjugated oxaliplatin prodrug (Oxa(IV)SA2) to obtain covalent organic polymer, THPP‐Oxa(IV)‐PEG, for synergistic efficacy of SDT and chemotherapy (Figure 8c). Treatment with THPP‐Oxa(IV)‐PEG and US irradiation (2 W, 40 kHz, 30 min) were most effective in inhibiting tumor proliferation, attributing to its high potential in triggering antitumor immune response. Synergistic integration enhanced targeted ICDs, accompanied by the effective increase of CRT and HMGB1 release (Figure 8c,d). Subsequently the DC maturation ratio of lymph nodes adjacent to the tumors was upregulated, and the percentage of intra‐tumoral CD3^+^CD8^+^ T cells were also increased remarkably, which contributed to the reversal of immunosuppressive TME and ultimately suppressed the tumor growth of CT26 tumor‐bearing mice (Figure 8c,e) with low systemic toxicity.^[^ ^119^ ^]^ Similarly, the chemotherapeutic drug, docetaxel (DTX), was encapsulated into a Rhein‐chondroitin sulphate‐based nanosystem (DTX/C‐NPs) to treat A549 tumor‐bearing mice by the synergistic therapy of chemotherapy and SDT (US parameters: 1.2 W cm^−2^, 3 min). The results showed that this synergistic treatment decreased the percentage of M2 macrophages, and successfully inhibited tumor growth and metastasis.^[^ ^120^ ^]^ Together, these studies demonstrated that SDT‐derived bimodal synergistic therapy could enhance ICD‐induced antitumor immunity, and achieve minimal damage to normal tissues but maximized efficiency, providing a new strategy for cancer treatment. Due to the clear composition, convenient synthesis and good biocompatibility of nanoparticles, these SDT‐derived bimodal synergistic therapy strategies have great prospects in future clinical translation, especially in radiotherapy and chemotherapy, and have been widely used in clinical practice.

#### 3.2.2 SDT‐Induced ICD and ICB Bimodal Therapy

**Figure 9:**
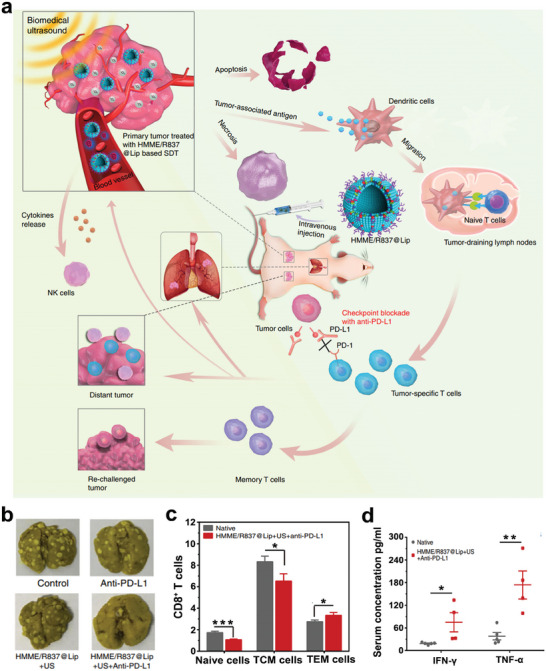
a) Schematic depiction of antitumor immune response induced by combination therapy of HMME/R837@Lip based SDT and checkpoint blockade for cancer immunotherapy. b) The analysis of pulmonary metastases in different groups. c) The proportions of naïve cells, TCM cells and TEM cells in CD8^+^ T cells. d) The serum concentrations of IFN‐γ and TNF‐α after different treatments. Reproduced with permission.^[^ ^122^ ^]^ Copyright 2019, Nature Publishing Group. * *p*< 0.05, ** *p*< 0.01, *** *p*< 0.001.

Although anti‐CTLA4 and PD‐L1/PD‐1 treatments achieve remarkable success in patients with various types of hematologic malignancies, insufficient T cell infiltration in hypoxic solid tumors limits their anticancer effects.^[^ ^121^ ^]^ Researchers proposed to combine SDT with ICB therapy to improve the antitumor efficacy. As shown in **Figure** **9**a, novel nanosonosensitizers (denoted as HMME/R837@Lip) were successfully synthesized, wherein liposomes served as the delivery vehicles to organic sonosensitizer molecules, hematoporphyrin monomethyl ether (HMME), and immune adjuvant R837. Notably, in vivo investigations have demonstrated that HMME/R837@Lip‐mediated SDT (US parameters: 1.0 MHz, 1.5 W cm^−2^, 50% duty cycle, 5 min) cooperated with anti‐PD‐L1 treatment could not only suppress the primary tumors, but also significantly inhibit the growth of distant tumors. While single treatment with anti‐PD‐L1 exhibited little inhibitory effect on primary and distant tumors, and SDT treatment alone could only suppress the growth of primary tumors. Furthermore, combination treatment with SDT and anti‐PD‐L1 substantially suppressed pulmonary metastasis (Figure 9b). In bilateral CT26‐bearing tumor models, the combination therapy achieved a similar tumor‐inhibition efficiency and produced long‐term immune memory effects, leading to an obvious transfer of native and central memory (TCM) CD8^+^ T cells towards effector memory T cell (TEM) phenotype (Figure 9c) and upregulation of serum TNF‐α and IFN‐γ levels (Figure 9d), implying the activation of systemic antitumor immunity.^[^ ^122^ ^]^ Considering that all major components in engineered nanosonosensitizers have been approved by the FDA, this approach holds significant promise as a viable therapeutic modality for cancer treatment.

For better immune sensitization, a SDT‐nanovaccine platform, cMn‐MOF@CM, was synthesized by combining immune adjuvant CpG and manganese porphyrin organic framework (Mn‐MOF) and subsequent coating by cell membranes originated from OVA‐overexpressing melanoma B16 cells. DAMPs derived from SDT‐induced ICD and OVA together exhibited vaccine‐like function with CpG, triggering a strong antitumor immune response, thereby induced the tumor immunosensitization to anti‐PD‐1 therapy in the treatment of malignant melanoma. In accordance with expectation, cMn‐MOF@CM‐mediated synergistic therapy of SDT (US parameters: 1.0 MHz, 1.5 W cm^−2^, 50% duty cycle, 5 min) and anti‐PD‐1 caused a systemic immune response and long‐term immune memory function, effectively preventing tumor growth and recurrence.^[^ ^123^ ^]^ Consequently, SDT‐induced ICD‐based antitumor immune response has the ability to augment the efficiency of ICB therapy, which can amplify systemic antitumor immunity, and stimulate memory immunity. This means may become the mainstream of antitumor therapy in the future.

### 3.3 SDT‐Derived Multimodal Synergistic Therapy

#### 3.3.1 Multimodal Synergistic Therapy to Enhance ICD‐Induced Antitumor Immune Response

**Figure 10:**
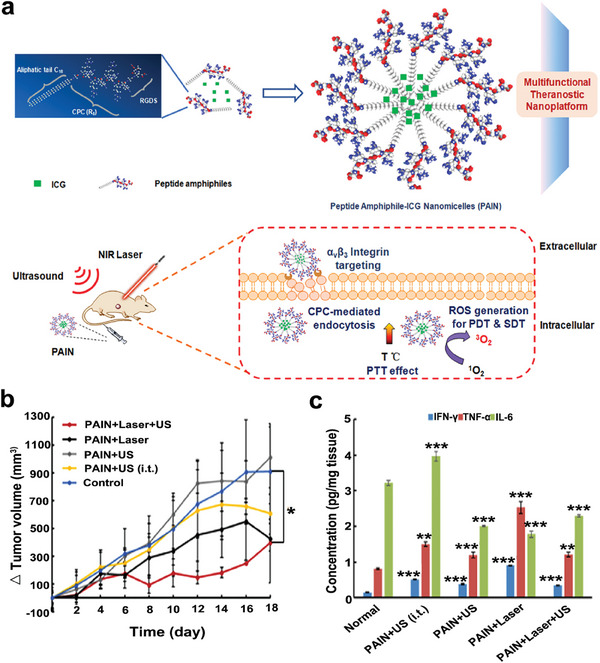
a) A schematic illustration of the PAIN composition and the combination therapy of SDT, PDT, and PTT for treating breast tumor‐bearing mice. b) The variations of tumor volume in mice with different treatments. c) The expression levels of pro‐inflammatory cytokines after various treatment. Reproduced with permission.^[^ ^125^ ^]^ Copyright 2020, Elsevier. * *p*< 0.05, ** *p*< 0.01, *** *p*< 0.001.

Due to the complex mechanism of tumor development, dual‐modal therapy containing SDT may not be capable to completely eradicate tumors and inhibit tumor recurrence, the exploration of multimodal therapy exhibits great prospects. Considering that the ICD‐induced immune response is promising, several studies have designed the therapy strategy for triple modalities to amplify the synergistic effect of ICD and further enhance the antitumor effect. Hence, a multifunctional phase‐transition nanoplatform (termed as OI_NPs) loaded with indocyanine green (ICG), oxaliplatin (OXP), and perfluoropentane (PFP), was successfully constructed for augmented therapeutic efficiency and immunological effect of chemotherapy, PDT, and SDT (PDST). Compared to other treatment groups, more ROS generation was observed in the OI_NPs + PDST group, leading to apparent apoptosis of ID8 cells. After treated with OI_NPs + PDST (1.5 W cm^−2^ 808 nm laser for 2 min and 1.0 W cm^−2^ US irradiation for 1 min), the CRT exposure on the cell surface of ID8 cells was evidently upregulated, and the expression level of cytoplasmic HMGB1 and the release of HMGB1 in the supernatant (S‐HMGB1) also increased, indicating that this multimodal synergistic therapy could enhance ICD.^[^ ^124^ ^]^ To construct the ideal strategy of using light/sound as the most natural methods to eliminate tumors, peptide amphiphile‐ICG nanomicelles (PAIN) were synthesized by the self‐assembly of the engineered peptide amphiphiles with ICG. The established multifunctional nanoplatform achieved the combination therapy of SDT, PDT, and PTT under the real‐time guidance of photoacoustic and ultrasonic bi‐modal imaging (**Figure** **10**a). As shown in Figure 10b, PAIN + laser (808 nm, 1.5 W cm^−2^, 3 min) + US (1 MHz, 2.4 W cm^−2^, 50% duty cycle, 5 min) revealed the best efficacy of tumor inhibition among different treatment groups, indicating the synergistic effect of SDT, PDT, and PTT. To elucidate the immunoregulatory effect of the combination therapy, the concentrations of pro‐inflammatory cytokines, including IFN‐γ, TNF‐α, and IL‐6, were analyzed. The results showed that IFN‐γ and TNF‐α levels were significantly elevated in all treatment groups compared to the control group, suggesting the presence of direct cell‐killing and anti‐angiogenic mechanisms Moreover, TNF‐α‐induced necrosis may coincide with IFN‐γ‐activated vascular degeneration. In the PAIN + US, PAIN + laser, and PAIN + laser + US groups, IL‐6 level was relatively lower, suggesting fewer side effects and a favorable prognosis (Figure 10c). H&E images of main organs confirmed no obvious damage to normal tissues.^[^ ^125^ ^]^ Based on these studies, it is hoped that early diagnosis and effective treatment through PDT/SDT could be realized via successful medical translations, potentially providing an alternative to traditional chemotherapy or radiotherapy.

#### 3.3.2 Multimodal SDT‐Induced ICD and ICB Therapy

**Figure 11:**
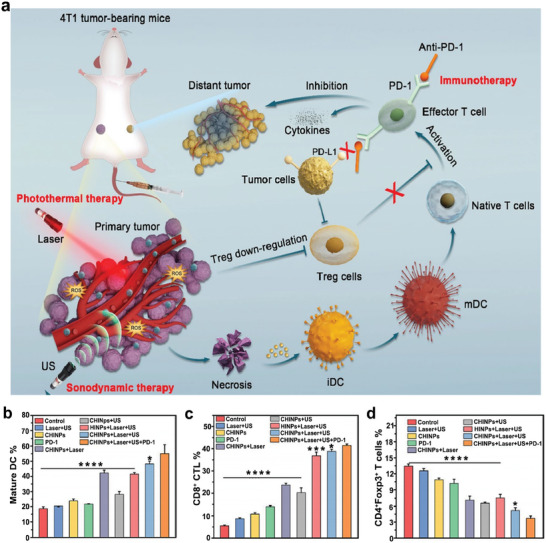
a) Schematic of CHINPs synthesis and the synergistic therapeutic effect of PTT, SDT, and anti‐PD‐1. b–d) The levels of DC maturation, CD8^+^ CTL and Treg cells in 4T1 tumor bearing mice with different treatments. Reproduced with permission.^[^ ^126^ ^]^ Copyright 2022, BioMed Central. * *p*< 0.05, *** *p*< 0.001, **** *p*< 0.0001.

Considering that ICD‐induced antitumor immune response and ICB therapy may have the potency of inducing antitumor memory immunity to prevent metastasis and recurrence, multimodal synergistic therapy of ICD‐induced antitumor immunity and ICB therapy have been proposed. Based on this, 4T1 cancer cell membranes‐biomimetic nanoparticles (CHINPs), loaded with the sonosensitizer HMME and the photothermal transduction agent superparamagnetic iron oxide (SPIO), were designed to allow nanoparticles accumulate in the tumor area and synergistically enhance SDT and PTT. SDT overcomes the inherent weakness of PTT in targeting deeper tissues, and PTT enhances SDT by increasing tumor blood flow and oxygen generation. A triple protocol was then constructed in combination with PD‐1 checkpoint blockade (**Figure** **11**a). The triple therapeutic strategies almost completely eradicated the primary tumors completely, and was significantly more effective than single SDT, PTT, ICB, and combination of SDT and PTT. Furthermore, the triple therapy strategy (laser parameters: 808 nm, 2.0 W cm^−2^, 10 min; US parameters: 1 MHz, 2.0 W cm^−2^, 5 min) significantly eliminated the distant tumors that were considered metastatic by promoting DC maturation, activating CD8^+^ cytotoxic lymphocytes, and downregulating Treg cells (Figure 11b–d).^[^ ^126^ ^]^ Therefore, triple therapy strategies may be the state‐of‐the‐art routes to remove tumors and reduce the recurrence and metastasis.

**Figure 12:**
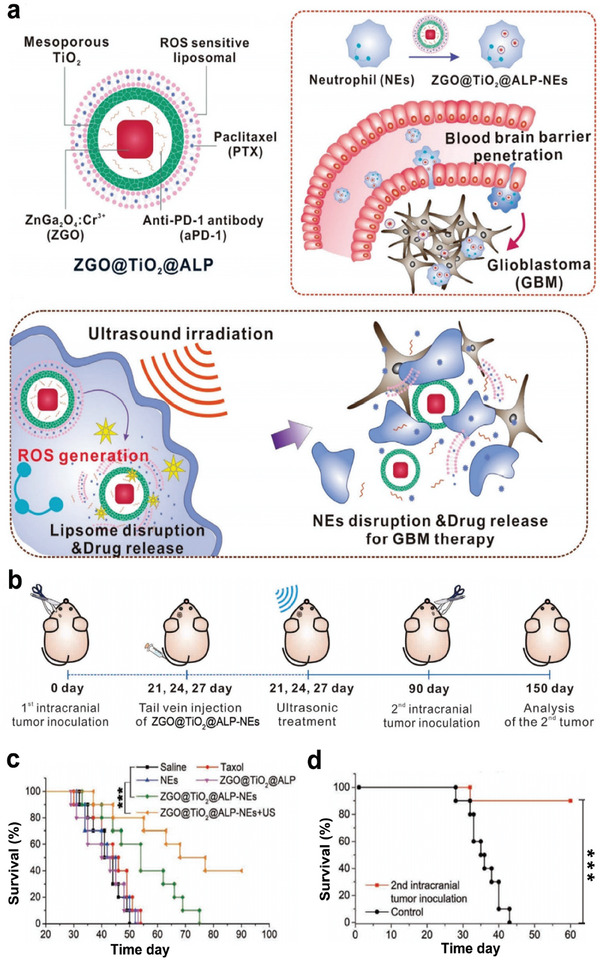
a) Illustration of ZGO@TiO_2_@APL synthesis and the mechanism of US amplified chemo/immuno glioblastoma therapy. b) Diagram of the experimental scheme. c) Survival curves of GL261 tumor‐bearing mice with different treatments. d) The survival analysis of glioblastoma rechallenged survivors. Reproduced with permission.^[^ ^127^ ^]^ Copyright 2021, Wiley‐VCH. *** *p*< 0.001.

Additionally, as shown in **Figure** **12**a, a neutrophil‐delivered nanosensitizer (ZGO@TiO_2_@ALP) composed of the luminescent phosphor ZnGa_2_O_4_:Cr^3+^(ZGO), TiO_2_, anti‐PD‐1 antibody, and paclitaxel (PTX), was successfully fabricated to achieve drug delivery through BBB in combination with SDT, chemotherapy, and ICB therapy for glioblastoma treatment. Glioblastoma models were established by intracranial inoculation of GL261 tumor cells, and rechallenged tumor models were constructed through intracranial inoculation of GL261 tumor cells on the other side of the brain of surviving mice in the ZGO@TiO_2_@ALP‐NEs + US group, at after initial glioblastoma implantation for 90 days (Figure 12b). Under US irradiation (1.5 MHz, 1.5 W cm^−2^, 5 min), ZGO@TiO_2_@ALP could not only eradicate the primary tumors and inhibit the formation of metastasis, thereby significantly improving survival (Figure 12c), but also prevent tumor recurrence (Figure 12d), which illustrates that the combined strategy triggers antitumor memory immunity.^[^ ^127^ ^]^ This multifunctional nanoplatform can make up for the defects of chemotherapy and anti‐PD‐1 therapy, which is both effective and low toxicity. Since chemotherapy and PD‐1 antibody have been widely used in clinical treatment, this therapeutic schedule may solve many limitations of current cancer therapy, and has a broad application prospect.

Together, these findings suggest that multimodal synergistic therapy could improve ICB efficiency through ICD‐induced immune sensitization, then trigger very strong antitumor immunity to reverse immunosuppressive TME, almost completely eradicating the primary tumor, and effectively preventing tumor metastasis and recurrence. Therefore, this multimodal synergistic therapy provides an excellent treatment strategy for tumor therapy, and may become the mainstream of antitumor therapy in the future.

## 4 Focused Ultrasound

### 4.1 FUS‐Mediated Monotherapy

#### 4.1.1 Modulating Immune Response via FUS‐Induced Thermal Effects

FUS is a non‐invasive, non‐ionizing technology for depositing focal energy into tissues with less than millimeter precision. In particular, HIFU has been widely applied in the clinical treatment of different tumors. Similar to conventional US, FUS has two main therapeutic effects on tissues: thermal and mechanical effects. The temperature of the thermal effect is related to the energy deposited. Normally, HIFU utilizes a high deposition energy (> 55 °C) to elicit thermal ablation.^[^ ^43^ ^]^ In addition to the immunomodulatory processes of wound healing response after ablation, HIFU‐induced thermal ablation may lead to coagulative necrosis with subsequent release of TAAs and a variety of bioactive molecules, such as DAMPs, which thus result in the activation of innate and adaptive immune responses.^[^ ^47^ ^]^ Given that unexpected tissue damage, accidental burns and pain may occur during HIFU application, and that some studies have reported that coagulative necrosis induced by HIFU treatment impaired the release of immunostimulatory molecules in TME, FUS with a low dose of deposition energy (< 55 °C) was proposed, such as low‐intensity focused ultrasound (LIFU).^[^ ^44^, ^128^, ^129^, ^130^, ^131^ ^]^ Studies have demonstrated that FUS‐induced hyperthermia with lower deposition energy doses could increase cellular permeability and further facilitate nanoparticle delivery.^[^ ^47^ ^]^ In addition, a study showed that although LIFU‐induced thermal effect could not directly induce tumor cell death, LIFU treatment (3 W, 5 min) could upregulate the level of DAMP signal, heat shock protein 70 (HSP70), and promote the activation of CD4^+^ T cells. The pretreatment of LIFU potentially led to the creation of a robust immunogenic response, stimulated by HIFU ablative therapy. This induced tumor death, offered protection against metastasis, and ensured a prolonged period of recurrence‐free survival.^[^ ^132^ ^]^ Therefore, FUS‐mediated thermal effects have the potential to modulate immunity, possibly leading to enhanced effectiveness in eliminating primary tumors and preventing distant metastases, especially when combined with an ablative approach. Considering the widespread use of FUS in clinical practice, this strategy holds considerable promise in clinical translation.

Additionally, increasing evidence has validated the critical role of tumor‐derived extracellular vesicles (EVs) in the tumor immune system, causing immunosuppressive and immunostimulating effects.^[^ ^133^ ^]^ Many oncology treatment interventions have been shown to increase the release of tumor‐derived EVs, such as chemotherapy,^[^ ^134^ ^]^ PDT,^[^ ^135^ ^]^ and irradiation.^[^ ^136^ ^]^ To investigate the effects of FUS‐mediated hyperthermia on EVs, a study showed that mouse glioma cells were treated with FUS in vitro, and found that FUS‐mediated hyperthermia effect could not only significantly increase the release of glioma‐derived EVs (GEVs), but also alter the proteomic profile of these GEVs. After treatment with FUS‐induced hyperthermia effect, several markers associated with cancer progression and drug resistance were down‐regulated, such as major vault protein (MVP), calumenin (CALU), and heat shock 70 kDa protein 5 (HSPA5). While complement C5 was upregulated after FUS treatment, indicating inflammation caused by hyperthermia. Furthermore, by coculturing mouse DC2.4 dendritic cells with GEVs, it was found that naïve GEVs inhibited the expression of IL‐12p70, an important regulator of DC maturation. While FUS‐treated GEVs promoted significant upregulation of IL‐12p70 production in DCs. This study partly explained the underlying mechanism by which FUS regulated antitumor immune response.^[^ ^137^ ^]^ This study provided evidence for the underlying mechanism of the relationship between FUS and the tumor immune system, promising to monitor responses to FUS using GEV‐related biomarkers.

#### 4.1.2 Enhancing Thermal Effects Induced Antitumor Immune Response

**Figure 13:**
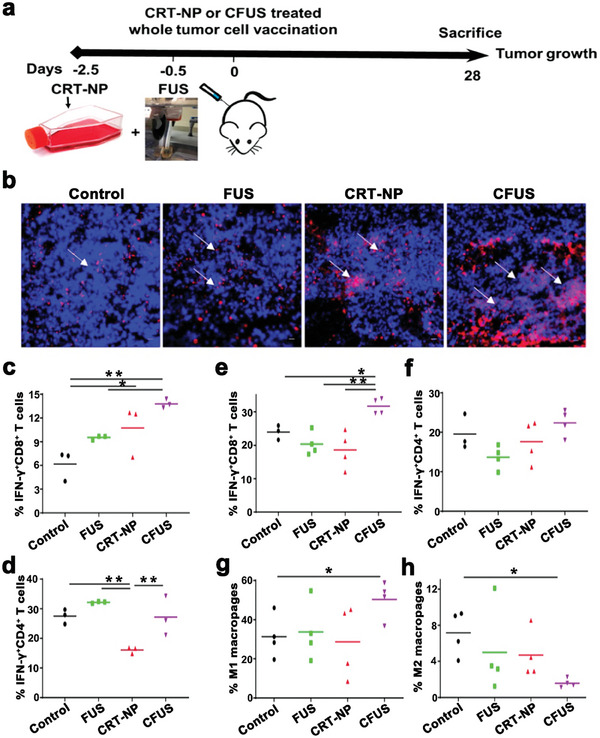
a) Experimental schedule for the combination therapy of CRT‐NPs with FUS against melanoma. b) Representative immunofluorescence images of CRT in B16F10 melanoma tumor sections. c,d) The populations of IFN‐γ^+^CD8^+^ T cells and IFN‐γ^+^CD4^+^ T cells in tumors with different treatments. e–h) The populations of IFN‐γ^+^CD8^+^ T, IFN‐γ^+^CD4^+^ T cells, M1, and M2 macrophages in spleens with different treatments. Reproduced with permission.^[^ ^138^ ^]^ Copyright 2020, IVYSPRING. * *p*< 0.05, ** *p*< 0.01.

Although the FUS‐induced thermal effect has the potency to modulate immunity and activate innate and adaptative immune responses, this immunomodulatory response is considered too weak to produce effective antitumor immunity. Numerous nanoplatform have been designed and fabricated to enhance the immune effects caused by FUS. A novel type of calreticulin‐nanoparticle (CRT‐NP) was reported to enhance ICD and synergize with FUS (5 Hz, 6 W, 50% duty cycle, 15 min, 42~45 °C) for achieving systemic antitumor effects (**Figure** **13**a). Compared to FUS/CRT‐NP alone, CFUS had higher CRT expression (Figure 13b), followed by increased MHCII expression on DCs, CD3^+^ T cell infiltration, and tumor suppression of the M1 macrophage population, indicating the activation of the immune system. The tumor growth curves revealed that the antitumor effect of CFUS was superior to that of FUS/CRT‐NP alone. Furthermore, the T cell phenotype in tumors was analyzed, and it was shown that CFUS treatment upregulated the population of CD8^+^/CD4^+^ T cells expressing IFN‐γ. Intriguingly, the population of IFN‐γ^+^CD4^+^ T cells was upregulated under FUS, indicating the potential of FUS to modulate immunity (Figure 13c,d). The concentrations of IL‐1β and TNF‐α in tumors also increased. Among all the groups, after treatment with CFUS, the population of spleen CD8^+^/CD4^+^ T cells expressing IFN‐γ and M1 macrophages were the highest, while the percentage of M2 in the spleen was significantly downregulated (Figure 13e–h). In the study of the tumor rechallenge model, it was observed that FUS alone could inhibit tumor growth to some extent, but the effect was relatively insignificant. On the other hand, CFUS demonstrated a significant ability to inhibit tumor recurrence. Furthermore, this suggested that CFUS could trigger a systemic immune response, an effect that proved to be more effective than FUS/CRT‐NP alone. Notably, CFUS treatment also enhanced the expression of PD‐1 on CD3^+^CD8^+^ T cells, indicating that adaptive resistance has emerged after CFUS treatment, and the inclusion of anti‐PD‐1 therapy can further improve antitumor efficiency.^[^ ^138^ ^]^

**Figure 14:**
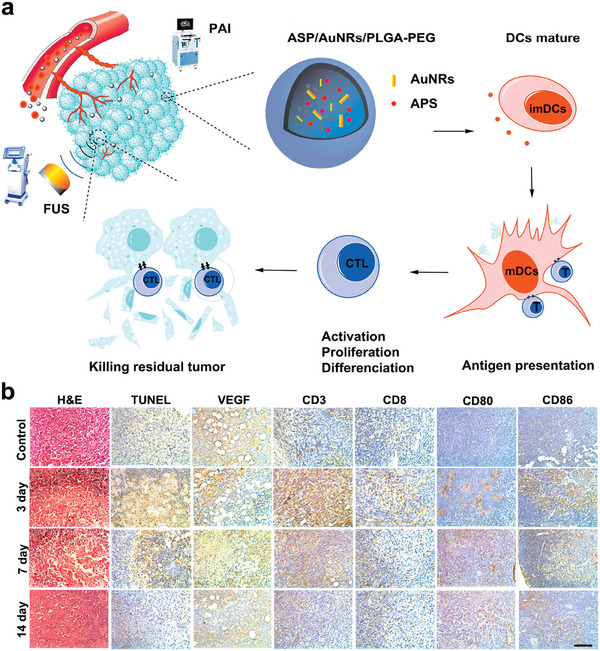
a) Schematic illustration of synthetic APS/AuNR/PLGA‐PEG NPs. b) H&E staining, TUNEL staining, and Immunohistochemical staining of positive expression of tumor‐infiltrating VEGF, CD3^+^, CD8^+^, and lymphoid‐infiltrating CD80^+^, CD86^+^ in the control group (0 day) and APS/AuNR/PLGA‐PEG NPs + FUS group at 3 days, 7 days, and 14 days. Reproduced with permission.^[^ ^141^ ^]^ Copyright 2020, Dove Medical Press.

Since astragalus polysaccharides (APs) has shown immunomodulatory effects and antitumor efficacy,^[^ ^139^, ^140^ ^]^ a multifunctional nanoplatform (APS/AuNR/PLGA‐PEG) was synthesized to boost FUS‐induced immune effect by encapsulating APs and gold nanorods (AuNRs) in PEGylated poly (D, L‐lactide‐co‐glycolide; **Figure** **14**a). The results revealed that on the 3rd day after HCC ablation treatment (FUS parameters: 5 W, 10 s), compared to PBS + FUS, APS/AuNR/PLGA‐PEG NPs + FUS significantly upregulated the levels of serum pro‐inflammatory cytokines, including TNF‐α and IFN‐γ, and increased infiltration of CD3^+^CD8^+^ T cells and mature DCs (CD80^+^CD86^+^) in tumors, suggesting that APS/AuNR/PLGA‐PEG NPs + FUS had excellent synergistic performance in enhancing immune regulation. Vascular endothelial growth factor (VEGF) and terminal deoxynucleotidyl transferase‐mediated nick end labeling (TUNEL) immunohistochemical staining confirmed the thermal ablation effect of APS/AuNRs/PLGA‐PEG NPs under FUS treatment (Figure 14b).^[^ ^141^ ^]^

#### 4.1.3 Modulating Immune Response via Mechanical Effects Induced by FUS

In addition to thermal effects, FUS could trigger mechanical effects, including radiation force, increased pressure, and cavitation effects, which can improve the permeability of cell membrane, thereby increasing the uptake of nanoparticles in the target tissues. MRI‐guided pulsed focused ultrasound (pFUS, 589.636 kHz, 1% duty cycle, 5 min) combined with US contrast agent microbubbles has been shown to cause transient destruction of the BBB in targeted brain regions, facilitating the delivery of molecules to the parenchyma. Meanwhile, significant molecular changes have taken place in the brain. After pFUS+MB treatment, the expressions of DAMP signal (HSP70), proinflammatory factors, including TNF‐α, IFN‐γ, and IL‐1β, as well as chemotactic factors, including granulocyte macrophage colony‐stimulating factor (GM‐CSF), and macrophage inflammatory protein 3 alpha (MIP3α) were significantly increased, following with an innate immune response, characterized by the infiltration of CD68^+^ macrophages in tumor These changes might be attributed to the cell damage caused by pFUS, and further lead to a temporal progression of systemic inflammatory cytokines.^[^ ^142^ ^]^ In addition to the changes in cytokines, FUS has also been proven to promote homing of various immune cells, including CD8^+^ CTLs, DCs, NK cells, neutrophils, and macrophages, with the assistance of microbubbles.^[^ ^143^, ^144^, ^145^, ^146^, ^147^ ^]^

Additionally, HIFU could initiate mechanical ablation using very short and high‐intensity pulses. These high‐pressure waves cause mechanical damage to tissues at the subcellular level by altering the composition of gases in tissues.^[^ ^148^ ^]^ S. Peng compared the therapeutic efficiency of thermal ablation and mechanical ablation in rabbits with liver tumors. It was found that mechanical ablation could lead to controlled damage to rabbit liver tumors, while thermal ablation might result in incomplete ablation.^[^ ^149^ ^]^ Mechanical ablation has been demonstrated to elicit the release of DAMPs, subsequently stimulate APCs, increasing the secretion of IL‐12 in DCs, and TNF‐α in macrophages. The stimulatory effect of mechanical HIFU (1.1 MHz, 3% duty cycle, 3 s) was higher than that of thermal HIFU (1.1 MHz, 30% duty cycle, 5 s). This superior performance is linked to the fact that thermal HIFU may yield an incomplete release of heat‐resistant danger signals such as ATP, while it simultaneously possesses the ability to denature and render heat‐sensitive danger signals like HSP60 inactive, along with endogenous molecule‐degrading enzymes like ATPases. On the flip side, mechanical HIFU, despite its minor thermal effect, exhibits a strong propensity to disrupt cell membranes, which could lead to a more extensive release of an array of endogenous danger signals. However, it should be taken into account that some of these signals could be broken down by endogenous molecule‐degrading enzymes released at the same time.^[^ ^150^ ^]^ In recent years, HIFU has been successfully employed in clinics to treat various types of cancer. However, in its current state, HIFU therapy is inadequate in addressing metastatic or even residual cells at the primary tumor site. This limitation is in part due to the fact that current HIFU therapy target thermal ablation of tumors almost exclusively, largely ignoring the biological impacts that can be generated by the mechanical stress applied by FUS in tissues. By investigating how tumor tissue responds to FUS‐induced mechanical stress, and by strategically combining HIFU‐induced antitumor immunity and thermal ablation, the overall quality and effectiveness of HIFU cancer treatment may be substantially improved in the future.

### 4.2 FUS‐Derived Synergistic Therapy

#### 4.2.1 Synergistic Therapy Mediated by Thermal Effects

**Figure 15:**
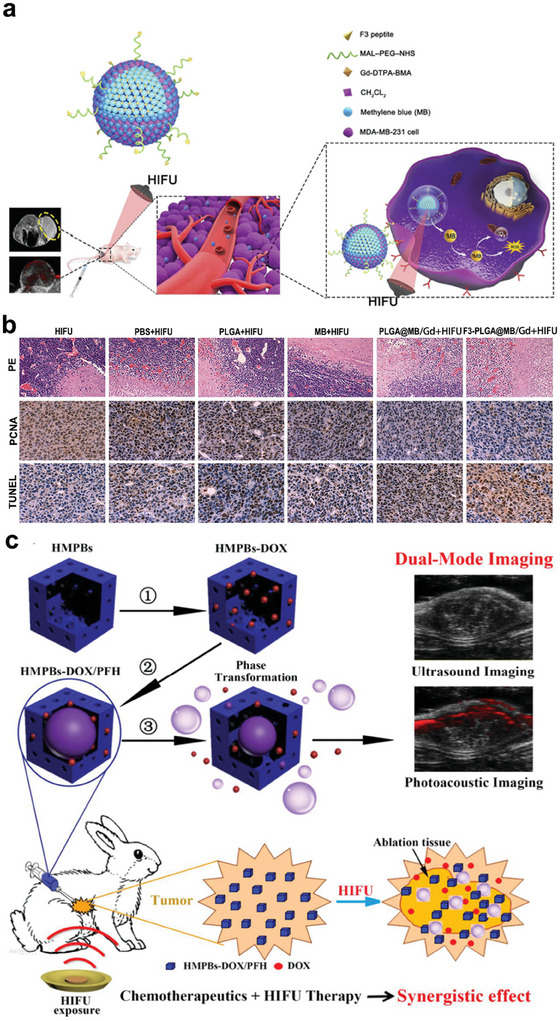
a) Schematic diagram illustrating the synthesis of F3‐PLGA@MB/Gd nanoparticle and the joint application of HIFU and SDT for accurate diagnosis and treatment of breast cancer. b) H&E staining (magnification, 100×), PCNA staining (magnification, 400×) and TUNEL staining (magnification, 400×) of tumor tissues after different treatments. Reproduced with permission.^[^ ^152^ ^]^ Copyright 2019, Dove Medical Press. c) Schematic diagram of the nanoplatform (HMPBs‐DOX/PFH) for imaging and tumor therapy. Reproduced with permisssion.^[^ ^50^ ^]^ Copyright 2016, IVYSPRING.

Although HIFU is widely explored as a non‐invasive treatment for solid tumors, HIFU ablation is less efficient in treating deep‐seated large tumors with insufficient blood supply, leading to incomplete HIFU ablation.^[^ ^148^, ^151^ ^]^ To improve efficiency, a multifunctional F3‐PLGA@MB/Gd nanosystem, loaded with sonosensitizer, methylene blue (MB), was synthesized to simultaneously achieve combination therapy (SDT and HIFU), dual‐modality imaging (photoacoustic and magnetic resonance imaging) and tumor targeting (**Figure** **15**a). As shown in Figure 15b, F3‐PLGA@MB/GD‐NPs + HIFU caused strong coagulative necrosis of tumor tissues, accompanied by a significant decrease in the level of proliferating nuclear antigens and an increase in apoptosis rate, which indicated that F3‐PLGA@MB/GD‐NPs combined with HIFU therapy (120 W, 3 s) could improve tumor ablation efficiency, and cause apoptosis of surrounding tumor cells, thereby killing residual tumor cells. The quantitative coagulation necrosis volume of the SDT and HIFU synergistic therapy was nearly 9 times larger than the HIFU alone.^[^ ^152^ ^]^

Given the widespread use of FUS and its potential to modulate immunity, several studies have proposed combination therapy with chemotherapy and FUS to improve the antitumor efficiency. As shown in Figure 15c, a multifunctional mesoporous prussian blue (HMPBs) nanoplatform (HMPBs‐DOX/PFH), embedded with chemotherapy drug DOX and perfluorohexane, was developed. This HMPBs‐DOX/PFH agent could not only enhance cavitation effect and coagulation necrosis upon exposure to HIFU (1.5 MHz, 42 °C, 20 min), but also accelerate the release of DOX, thereby enhancing chemotherapy. The low intensity of HIFU and local release of DOX could simultaneously reduce the side effects of HIFU and chemotherapy, including the damage towards normal tissues caused by high‐focused acoustic power during HIFU treatment, as well as the systematic side effects caused by chemotherapeutic drugs. Therefore, this synergistic therapy has great promise.^[^ ^50^ ^]^ In addition, the synergistic treatment of US‐mediated hyperthermia and DOX has been shown to significantly induce ICD, and systemic antigen cross‐presentation of APCs, including DCs and macrophages. These may partly explain the excellent antitumor efficiency of synergistic therapy of FUS and chemotherapy.^[^ ^153^ ^]^

Although CAR‐T cell therapy has achieved great success in the clinical treatment of hematological malignancies, there are still many limitations in the treatment of solid tumors. Due to the large amount of antigen overlap between solid tumors and normal tissues, especially under tissue injury/inflammation, it is difficult to distinguish between tumors and normal tissues, causing non‐tumor toxicity to normal tissues and threatening lives.^[^ ^154^, ^155^, ^156^, ^157^ ^]^ To precisely control the activation of CAR‐T cells, a FUS‐based approach was developed to convert ultrasonic signals into genetic and cellular activation via preparing heat‐shock‐protein promoter‐driven CAR‐T cells, enabling CAR‐T cells to exert specific cytotoxic effects in tumor‐restricted regions subjected to short‐pulsed FUS‐induced hyperthermia. This reversible FUS‐CAR‐T cell system could not only achieve highly effective therapy, but also prevent targeted extratumoral side effects, allowing optimal efficacy and controllable T cell exhaustion in the future.^[^ ^158^ ^]^ We believe that as sonogenetics continues to be refined and optimized, it will present a universally applicable technique that heralds a new era of direct and non‐invasive manipulation of genetically engineered cells using US.

#### 4.2.2 Synergistic Therapy Mediated by Mechanical Effects

**Figure 16:**
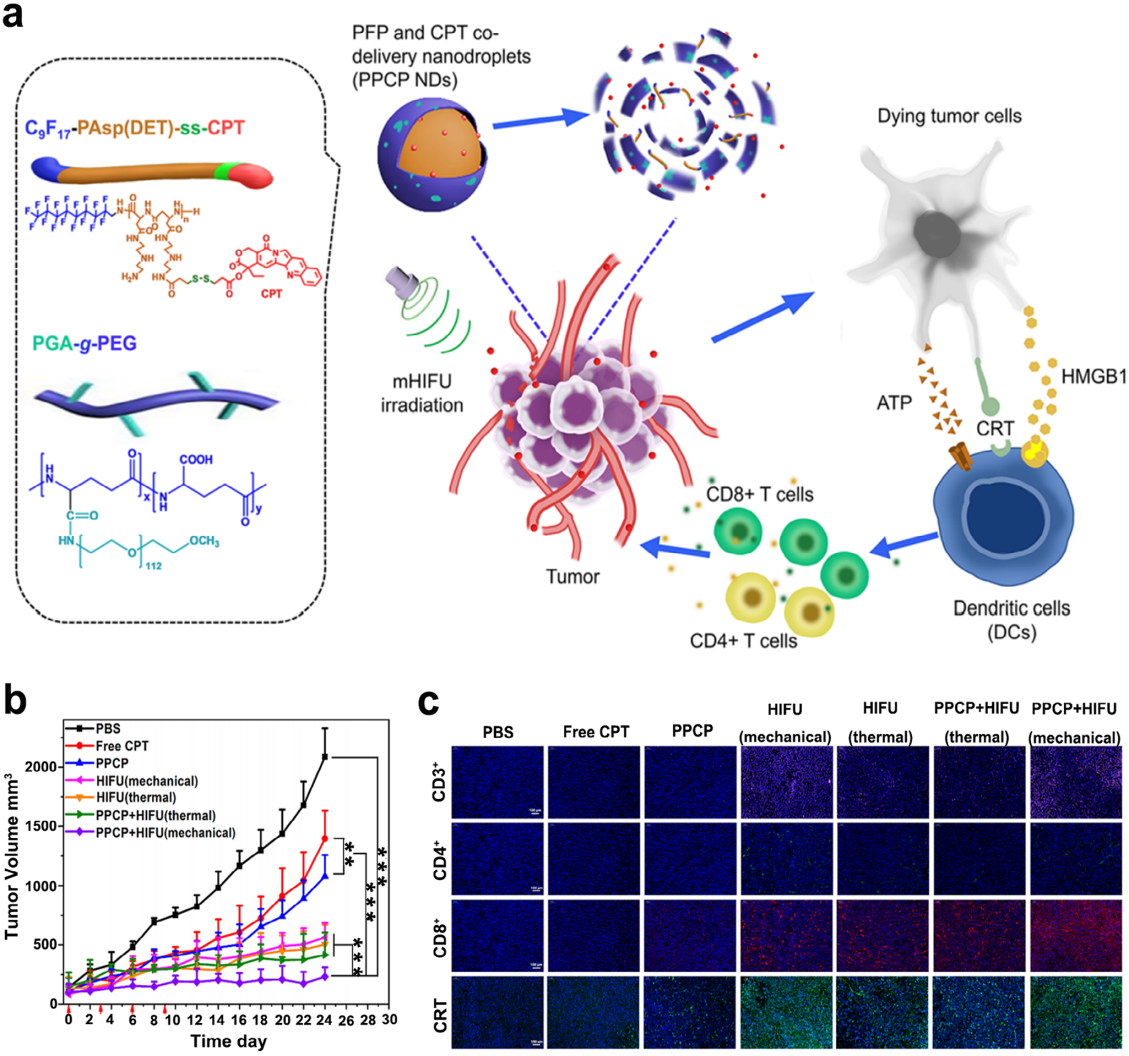
a) Schematic representation of the composition of PPCP NDs. b) The variations of tumor volume in mice with different treatments over time. c) Immunofluorescence analysis of the expressions of CRT, CD3^+^, CD4^+^, and CD8^+^ in tumor tissues post different treatments. Reproduced with permission.^[^ ^159^ ^]^ Copyright 2021, American Chemical Society. ** *p*< 0.01, *** *p*< 0.001.

Since FUS‐induced mechanical effects would elicit certain immune response, researchers believe that the synergistic effects of FUS‐induced mechanical effect with other treatments may have better antitumor efficiency. Based on this, nanodroplets (PPCP NDs) were constructed to combine mechanical HIFU ablation with chemotherapy to augment antitumor immunity by ultrasonic emulsification of a broad‐spectrum anticancer drug, camptothecin (CPT), and perfluoropentane (**Figure** **16**a). As shown in Figure 16b, PPCP + HIFU (mechanical, 3.5 MHz, 5 W, 20% duty cycle) achieved the best antitumor efficiency compared to other groups. Importantly, HIFU (mechanical) is superior to HIFU (thermal) in both combination therapy and monotherapy. To further investigate the mechanism, immunofluorescence analysis was performed, which showed that both PPCP and HIFU treatments elicited ICD with the increase of CRT, and subsequently enhanced the infiltration of CD3^+^, CD4^+^, and CD8^+^ T cells in tumors. Among all the groups, the levels of CRT, CD3^+^ T cells, CD4^+^ T cells, and CD8^+^ T cells were the highest in the PPCP + HIFU (mechanical) group (Figure 16c).^[^ ^159^ ^]^

Mechanical HIFU has been demonstrated to induce mechanical fractionation of tumors, which could effectively trigger the immunosensitization, reverse the immunosuppressive TME, and further augment the therapeutic effect of ICB therapy. A synergistic treatment of glioblastoma with mechanical HIFU ablation using perfluorocarbon (PFC) liquid filled silica microshells and PD‐1 checkpoint blockade was proposed. They found that the synergistic therapy of mechanical HIFU (1.1 MHz, 2% duty cycle, 2 min) with anti‐PD‐1 therapy exhibited significantly increased levels of CD8, CD45 as well as IFN‐γ, a marker for T cell activation, transforming the immune “cold” microenvironment into a “hot” microenvironment. Furthermore, tumor re‐challenge experiments in this study demonstrated that the host developed acquired immune memory for glioblastoma after synergistic treatment combined with mechanical HIFU ablation and PD‐1 checkpoint blockade.^[^ ^160^ ^]^ Since chemotherapy, HIFU and ICB have been widely used in the clinical treatment of tumors, their combination therapy may have great application prospects, especially mechanical HIFU.

## 5 Conclusion and Outlook

**Figure 17:**
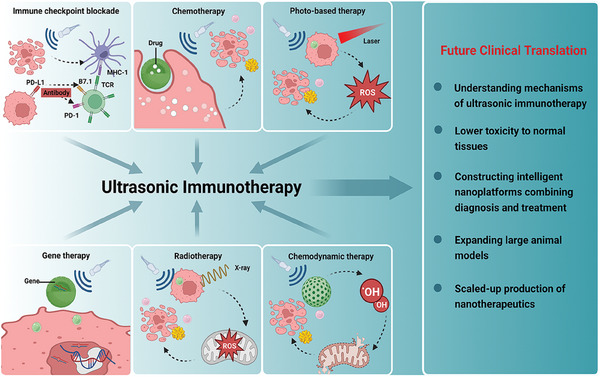
Summative scheme of synergistic therapy strategies based on ultrasonic immunotherapy and their further development for future potential clinical translation. (Created with bioRender.com)

In summary, the birth of US therapy technologies has innovated tumor treatment strategy, which can not only achieve good antitumor effect, but also induce immunological effects, which helps to regulate immunosuppressive TME. This review summarizes the effects of different therapeutic US therapy technologies, including UTMD/UTND, SDT and FUS on the tumor immune microenvironment and their main mechanisms (**Table** **1**). Generally, the most known immunomodulatory effects are based on US‐induced targeted tumor‐cell ICD, accompanied by the release of tumor‐associated antigens, which in turn promotes DCs maturation and further activates effector cells, including CD8^+^ CTLs and NK cells. Although these immune responses may not be effective in constructing antitumor immunity, they can induce tumor sensitization to other forms of immunity. Recent studies have demonstrated that this ICD‐associated immunogenicity is more potent when driven by ROS‐based ER stress, rather than merely as a secondary or collateral effect of ER stress triggered by chemotherapeutic drugs such as mitoxantrone and doxorubicin. Furthermore, this targeted ROS‐based ER stress has been found to escalate the amounts of DAMPs.^[^ ^30^ ^]^ This suggests that SDT could potentially stimulate an amplified ICD‐related immune response rather than chemotherapy. Compared to other ROS‐based ER stress‐inducing therapies such as CDT, PDT, PTT, and radiotherapy, SDT causes less damage while providing superior tissue penetration. There are relatively scarce studies on the immunomodulatory capacity of UTMD/UTND compared to other therapies. However, given their high penetration and non‐invasiveness nature, they can be reused and possess significant application potential in the treatment of deep tumors. Hence, they hold considerable promise in effectively treating tumors. In addition, enormous advances in nanomaterials provide the possibility to improve the antitumor efficiency and immunological effects of therapeutic US, as well as therapeutic US‐derived bimodal and multimodal combination therapies. Admitting that synergistic therapies mediated by novel nanomaterials can reverse the immunosuppressive TME, construct a strong antitumor immunity, and then exhibit excellent tumor suppressive efficiency, there are still several critical issues to be solved in clinical translation (**Figure** **17**).

1. The mechanisms of UTMD/UTND, SDT and FUS remain largely unelusive, which may hinder their application. The US parameters currently used in studies are inconsistent, including the frequency, intensity, pulse repetition frequency, and duty cycle of US, duration, power, and pressure, and the US parameters applied to animal models must differ from those applied to humans, so the physical properties and subsequent biological effects of US therapy will be more or less different. Furthermore, different physical effects caused by therapeutic US therapy technologies also have different application values in tumor therapy, such as HIFU‐mediated thermal and mechanical ablation. Hence, the mechanism of US therapy needs to be further studied to provide theoretical guidance for optimizing US therapy.
2. Currently, most studies emphasize the value of UTMD/UTND, SDT, and FUS in tumor therapy with high penetration ability and non‐invasiveness, but few studies focus on their immunomodulatory capabilities. Immunity is very important throughout the entire course of tumors, including occurrence, progression, and outcome. Although most of the known immunomodulatory effects of therapeutic US therapy technologies are considered insufficient to effectively inhibit tumor growth, metastasis, and recurrence, this immunomodulatory effect can be augmented with the assistance of nanomaterials, and can synergistically enhance the antitumor immune response induced by other oncology therapies. Therefore, in‐depth study on the immunoregulatory effects of US will contribute to smart nanomaterials engineering and better combination therapy strategies.
3. Since US is noninvasive, especially UTMD/UTND and HIFU techniques have been applied in clinical practice, the key to the successful clinical translation of nanomaterials‐augmented US therapy is to design nanoparticles with low systemic toxicity. Although nanomaterials exhibit low toxicity in a relatively short period of time, due to their relatively large size and high stability, they may accumulate and remain in the body for a long time, resulting in long‐term toxicity to normal tissues. In particular, nanovaccine applications, such as US‐responsive nanovaccine, require long‐term retention of nanomaterials in vivo, so improving biocompatibility is a crucial step in nanomaterial design. In addition, nanotherapeutics with renal clearance may provide another strategy to reduce systematic toxicity.
4. Aiming at the complex mechanism of tumorigenesis and progression, bimodal and multimodal therapy strategies combining US therapy with other treatment modalities have been put forward, which sheds new light for cancer treatment. The realization of this synergistic therapy strategy depends to a large extent on the successful synthesis of multifunctional nanoparticles. In addition, it is highly desirable to establish nanoplatforms that integrate diagnostic and therapeutic functions to enable accurate cancer diagnosis and treatment guided by US imaging. However, with the improvement of nanoparticle capabilities, the design and synthesis process may become more complicated, boosting the cost of cancer treatment and hindering clinical translation. Consequently, it is of great significance to design intelligent nanoparticles with simplified synthesis methods and integrated functions.
5. Recently, small animal models and animal‐derived tumor cells are mostly used in experiments to investigate the therapeutic efficacy of nanomaterials, and they are very limited in interpreting their therapeutic effects in humans. To better understand actual therapeutic effects, large animal models and patient‐derived tumor cells and xenografts may be more appreciated. Therefore, the study of nanotherapeutics in large animal models should be expanded to facilitate the implementation of preclinical experiments.

**Table 1:** A summary of representative functional nanosystems for ultrasonic immunotherapy of tumors.

Therapeutic US technologies	Material names	Biomedical application	US parameters	Immunological effects	Reference
UTMD/UTND	UTNBs	Synergistic therapy	1 MHz, 1 W/cm^2^, 30s	Tumor cell lysis induced by cavitation effect, resulting in APC activation	[54]
	SNO‐HAS‐PTX	Drug delivery; Chemotherapy/gas therapy	1 MHz, 2 W/cm^2^, 5 min	Promoting the infiltration of intra‐tumoral CD4^+^/CD8^+^ T cells	[60]
	BRN	Drug delivery; ICB/ Radiotherapy	0.5 W/cm^2^, 3 min	Increasing blood‐brain barrier permeability and assisting anti‐PD‐1 therapy and radiotherapy	[76]
	OPR NPs	Drug delivery	40 kHz, 6 W/ cm^2^, 50% duty cycle, 5 min	An US‐responsive nanovaccine	[85]
	PLGA‐b‐PEG NPs	Gene delivery	1.8 MHz, 10 min^[^ ^92^ ^]^; 2.0 MHz, 2 min^[^ ^94^ ^]^	Upregulating the antitumor cytokines and inducing the CD8^+^ T cells infiltration in tumor region	[92, 94]
SDT	FA‐MnPs	SDT‐derived single mode therapy	1.0 MHz, 2.0 W/cm^2^, 50% duty cycle, 5 min	Actuating the ICD‐based antitumor immune response	[45]
	TiO2@CaP	Intensive SDT	3 MHz, 2.1 W, 20 min	Potentiated ICD‐based antitumor immune response	[98]
	MLipRIR NPs	Intensive SDT	1 MHz, 1.5 W/cm^2^, 50% duty cycle	Inducing severe ferroptosis and augmenting ICD	[100]
	RSL3@O_2_‐ICG NB	Intensive SDT	1.0 W/cm^2^, 30 s	Regulating the proportion of immune cells in the TME and improving the sensitivity of ferroptosis	[104]
	PCN‐224	Intensive SDT	1.0 MHz, 0.5 W/cm^2^, 50% duty ratio, 10 min	Depleting extracellular matrix of tumor tissues and implementing sonodynamic‐immunomodulatory pyroptotic strategy	
	CCM‐HMTNPs/HCQ	Intensive SDT	1 W/cm^2^, 30 s	Promoting autophagy of tumor cells and making them more sensitive to SDT	[109]
	Tf@IR820‐DHA	SDT/ CDT	0.4 W/cm^2^, 5 min	Enhanced ICD‐based antitumor immune response	[111]
	PIH‐NO	SDT/ Gas therapy	1.0 MHz, 1 W/cm^2^, 5 min	Amplified ICD‐induced immune response	[114]
	TAPP	SDT/ PTT	1.0 MHz, 1.0 W/cm^2^, 60% duty cycle, 5 min	Integrated diagnosis and treatment functions	[116]
	FA‐CLC/SPIO	SDT/ Radiotherapy	1 W/cm^2^, 20% duty cycle, 10 min	Promoting tumor‐cell apoptosis	[118]
	THPP‐Oxa(IV)‐PEG	SDT/ Chemotherapy	2 W, 40 kHz, 30 min	Targeted ICD and reversal of immunosuppressive TME	[119]
	DTX/C‐NPs	SDT/ Chemotherapy	1.2 W/cm^2^, 3 min	Decreasing the percentage of M2 macrophages	[120]
	HMME/R837@Lip	SDT/ ICB	1.0 MHz, 1.5 W/cm^2^, 50% duty cycle, 5 min	Obvious transfer of native and central memory CD8^+^ T cells to effector memory T cell phenotype	[122]
	cMn‐MOF@CM	SDT/ ICB	1.0 MHz, 1.5 W/cm^2^, 50% duty cycle, 5 min	ICD‐based antitumor immune response augmenting the efficiency of ICB therapy	[123]
	OI_NPs	SDT/Chemotherapy/PDT	1.0 W/cm^2^, 1 min	Augmented ICD‐induced antitumor immune response	[124]
	PAIN	SDT/ PDT/ PTT	1 MHz, 2.4 W/cm^2^, 50% duty cycle, 5 min	Achieving photoacoustic and ultrasonic bi‐modal imaging and favorable therapeutic effect at the same time	[125]
	CHINPs	SDT/ PTT/ ICB	1 MHz, 2.0 W/cm^2^, 5 min	Reversal of immunosuppressive TME	[126]
	ZGO@TiO_2_@ALP	Drug delivery; SDT/ Chemotherapy/ ICB	1.5 MHz,1.5 W/cm^2^, 5 min	Triggering antitumor memory immunity	[127]
FUS	CRT‐NP	FUS (thermal effect)	5 Hz, 6 W, 50% duty cycle, 15min	ICD‐based antitumor immune response	[138]
	APS/AuNR/PLGA‐PEG	FUS (thermal effect)	5 W, 10s	Excellent performance in enhancing immune regulation	[141]
	US contrast agent microbubbles (MB)	FUS (mechanical effect)	589.636 kHz, 1% duty cycle, 5 min	Inducing significant molecular changes in the brain	[142]
	F3‐PLGA@MB/Gd	HIFU (thermal effect)/ SDT	120 W, 3 s	Causing strong coagulative necrosis of tumor tissues	[152]
	HMPBs‐DOX/PFH	HIFU (thermal effect)/ Chemotherapy	1.5 MHz, 20 min	Systemic antigen cross‐presentation of APCs	[50]
	PPCP NDs	HIFU (mechanical effect)/ Chemotherapy	3.5 MHz, 5 W, 20% duty cycle	ICD‐based antitumor immune response	[159]
	Perfluorocarbon liquid filled silica microshells	HIFU (mechanical effect)/ ICB	1.1 MHz, 2% duty cycle, 2 min	Transforming the immune “cold” microenvironment into a “hot” microenvironment	[160]

With the continuous deepening of therapeutic US technology studies, and the remarkable progress of nanotechnology, there are great innovations in the field of tumor therapy, which has improved ultrasonic immunotherapy, regulated immunosuppressive TME, and further obtained excellent antitumor efficiency. Since nanomaterial‐based US therapy strategies remain more or less problematic, more multidisciplinary efforts are needed to achieve clinical translation of this novel therapy strategy.

## Conflict of Interest

The authors declare no conflict of interest.
